# Inhibition of Sec61-dependent translocation by mycolactone uncouples the integrated stress response from ER stress, driving cytotoxicity via translational activation of ATF4

**DOI:** 10.1038/s41419-018-0427-y

**Published:** 2018-03-14

**Authors:** Joy Ogbechi, Belinda S. Hall, Thomas Sbarrato, Jack Taunton, Anne E. Willis, Ronald C. Wek, Rachel E. Simmonds

**Affiliations:** 1 Department of Microbial Sciences, School of Biosciences and Medicine, University of Surrey, Guildford, Surrey GU2 7XH UK; 2 MRC Toxicology Unit, Lancaster Rd, Leicester, LE1 9HN UK; 3 Department of Cellular and Molecular Pharmacology, University of California, San Francisco, San Francisco, CA 94158 USA; 4 Department of Biochemistry and Molecular Biology, Indiana University School of Medicine, Indianapolis, IN 46202-5126 USA

## Abstract

Mycolactone is the exotoxin virulence factor of *Mycobacterium ulcerans* that causes the neglected tropical disease Buruli ulcer. We recently showed it to be a broad spectrum inhibitor of Sec61-dependent co-translational translocation of proteins into the endoplasmic reticulum (ER). An outstanding question is the molecular pathway linking this to its known cytotoxicity. We have now used translational profiling to better understand the reprogramming that occurs in cells exposed to mycolactone. Gene ontology identified enrichment in genes involved in cellular response to stress, and apoptosis signalling among those showing enhanced translation. Validation of these results supports a mechanism by which mycolactone activates an integrated stress response meditated by phosphorylation of eIF2α via multiple kinases (PERK, GCN, PKR) without activation of the ER stress sensors IRE1 or ATF6. The response therefore uncouples the integrated stress response from ER stress, and features translational and transcriptional modes of genes expression that feature the key regulatory transcription factor ATF4. Emphasising the importance of this uncoupled response in cytotoxicity, downstream activation of this pathway is abolished in cells expressing mycolactone-resistant Sec61α variants. Using multiple genetic and biochemical approaches, we demonstrate that eIF2α phosphorylation is responsible for mycolactone-dependent translation attenuation, which initially protects cells from cell death. However, chronic activation without stress remediation enhances autophagy and apoptosis of cells by a pathway facilitated by ATF4 and CHOP. Our findings demonstrate that priming events at the ER can result in the sensing of stress within different cellular compartments.

## Introduction

Mycolactone is a toxin produced by *Mycobacterium ulcerans*, the causative organism of Buruli ulcer^1^ (BU). All BU pathology is caused by this diffusible polyketide-derived compound acting remotely from the bacteria^2^. It is cytotoxic and immunosuppressive, inhibiting production of cytokines, chemokines and adhesion molecules^3^. The cellular effects of mycolactone can be attributed to its inhibitory effect on the Sec61 translocon^4^. Mycolactone inhibits co-translational translocation of secretory proteins, Type II transmembrane proteins (TMPs) and most Type I TMPs^5,6^, an essential early step in targeting most membrane and secretory proteins to the correct compartment^7^. Known Sec61 inhibitors include eeyarestatin I (ESI), CAM741/cotransin and decatransin^8^, and mycolactone can compete with cotransin for Sec61α binding, suggesting a similar interaction site^9^. However, mycolactone is more potent than all these, inhibiting protein translocation at nanomolar doses^4,9^. Despite great advances in our understanding of mycolactone function, the mechanistic linkage between translocation blockade and cell death by apoptosis^9,10^ has not been defined.

The integrated stress response (ISR) is a highly conserved adaptation to stress centred upon phosphorylation of the alpha subunit of eukaryotic initiation factor 2 (eIF2α)^11^. Four eIF2α kinases sense distinct stress conditions: HRI (EIF2AK1), PKR (EIF2AK2), PERK (EIF2AK3) and GCN2 (EIF2AK4). Phosphorylation of eIF2α (p-eIF2α) inhibits global translation, conserving cellular resources and facilitating reprogramming of gene expression. Simultaneously, p-eIF2α directs preferential translation of a subset of ‘stress response’ mRNAs, including ATF4, via a delayed translation reinitiation that allows ribosome scanning through inhibitory upstream open reading frames (uORF)^12^. ATF4 drives enhanced transcription of genes that collectively can alleviate stress^13^, including GADD34 (PPP1R15A), which targets PP1 for dephosphorylation of p-eIF2α in the feedback control of the ISR^14,15^. During chronic stress this pathway can also act to promote apoptosis, mediated by another ATF4 target; CHOP (DDIT-3)^16^.

The eIF2α kinase PERK is usually considered to be activated by endoplasmic reticulum (ER) stress, sensing disturbances in calcium homeostasis, redox status or protein load that compromise protein folding and assembly in this organelle^17^. PERK represents one arm of the Unfolded Protein Response (UPR), acting in conjunction with two other ER stress sensors, IRE1 and ATF6. Upon ER stress, IRE1 undergoes autophosphorylation and activation, resulting in cytosolic splicing of XBP1 mRNA^18–20^, whereas ATF6 transits from the ER to the Golgi where it is cleaved into its active form^21^. Both XBP-1 and ATF6 mediate their cellular effects as transcription factors that reprogramme gene expression.

Previously, we noted global changes to polysome profiles in cells after mycolactone exposure^4^. Here, we use ‘translational profiling’ to reveal pan-activation of the ISR without concurrent ER stress. Instead inhibition of Sec61 at the ER is sensed in the cytosol, leading to changes in translational and transcriptional control, autophagy and ultimately, cell death.

## Results

### Translational profiling identifies response to stress as an enriched gene ontology functional group

**Fig. 1:**
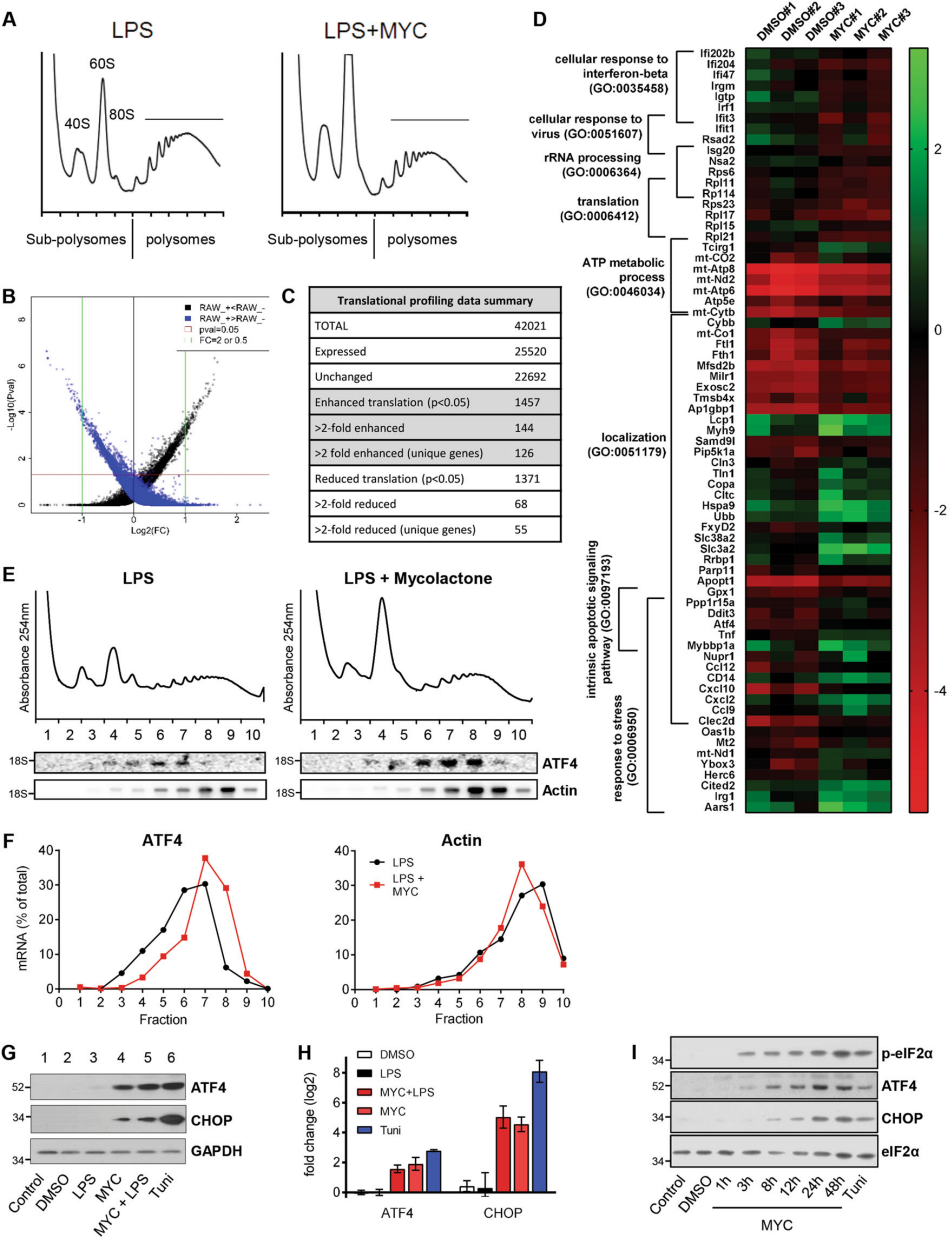
Translational microarray of cells exposed to mycolactone identifies ATF4 as translationally upregulated. **a**−**g** RAW264.7 cells pre-incubated for 1 h (mycolactone (MYC) or DMSO) then stimulated with 100 ng/ml LPS for 4 h or tunicamycin alone (Tuni). **a**−**e** Cell lysates were subject to polysome profiling and translational microarrays, as described in the text. **a** Mycolactone induces changes in polysome profiles. RNA purified from subpolysomal fractions (1–5) and polysomal fractions (6–10) was pooled and used in microarray analysis as described in Methods. **b** Scatter plot for probes in the microarray. Black and blue dots represent probes enriched in either polysomes or sub-polysomes respectively. Rank product analysis rules out changes in transcription and achieves a high validation rate for translationally regulated targets. **c** Summary of microarray data following translational profiling analysis as described in Methods. **d** Heatmap showing representative data for genes in eight significantly overrepresented gene ontology groups (*p* < 0.05), identified by PANTHER. **e** Northern blotting for transcripts in individual gradient fractions from LPS stimulated RAW264.7 cells, the migration of 18S rRNA is indicated; quantified in (**f**); *n* = 3 independent experiments. **g** Cell lysates were analysed by immunoblotting. **h** Relative fold change (ΔΔCt) for steady-state mRNA levels determined by one-step qRT-PCR on total RNA (Mean ± SEM, *n* = 3 independent experiments). **i** HeLa cells were treated as shown and lysates were analysed by immunoblotting. All immunoblots show the approximate migration of molecular weight markers in kDa. See also Figs. S1 and S2, Tables S1 and S2

Polysome profiling performed on activated macrophages (LPS-stimulated RAW264.7 cells) showed that, as previously described^4^, mycolactone reduced the peak area in polysomal fractions and increased that of sub-polysomal fractions (Fig. 1a). This pattern, indicative of repression of translation initiation, also occurs in the absence of LPS^4^ (Fig. S1A). However, the effect is weak compared to positive control tunicamycin, a pharmacological inducer of ER stress^12^ (Fig. S1B).

To better understand this reprogramming, we performed translational profiling, analysing genome-wide redistribution of gene transcripts in mycolactone-exposed cells^22,23^. This approach determines changes in the relative abundance of polysomal vs. sub-polysomal transcripts as a surrogate measure of translational efficiency^23^. Of probes that detected targets, approximately 5% were significantly altered between the different pools in response to mycolactone (*p* < 0.05, Fig. 1b, c, Tables S1 and S2; GEO: GSE103002).

Gene ontology (GO) analysis of unique genes with transcripts altered >2-fold (Fig. 1d) showed enrichment in sub-polysomes (reduced translation) of genes involved in interferon β responses, rRNA processing and translation. Those enriched in polysomes (enhanced translation) included genes involved in ATP synthesis, cellular response to stress and apoptosis signalling (Fig. 1d); in particular *ATF4*, *CHOP* and *GADD34* warranted further study, given the known cytotoxic effects of mycolactone.

### Induction of ATF4 and CHOP is a common feature of cells exposed to mycolactone

Northern blots confirmed redistribution of *ATF4* mRNA within polysome profiles (Fig. 1e; quantified in Fig. 1f, left). Mycolactone caused a strong induction of ATF4 protein expression (Fig. 1g) independent of cell activation (Fig. 1g, compare lanes 4 and 5). Induction of ATF4 expression was associated with a 3.9 ± 0.7-fold (*p *< 0.0001) increase in ATF4 mRNA (Fig. 1h). Thus, mycolactone increases both ATF4 mRNA and its translational efficiency, a mode of gene expression characteristic of the ISR consequent to phosphorylation of eIF2α.

CHOP, a known downstream target of ATF4^12^, was also induced by mycolactone, similarly independent of LPS (Fig. 1g). *CHOP* showed a large increase in steady-state mRNA level (25.4 ± 5.1 fold (*p *< 0.0001, Fig. 1h), suggesting ATF4-directed transcriptional induction accounts for a major portion of the observed increase in protein abundance.

Since induction of ATF4 and CHOP were independent of cell activation, we determined whether mycolactone induced ISR regulators in a hierarchical fashion in the more genetically tractable HeLa cells (Fig. 1i). As in RAW264.7 cells, we observed p-eIF2α 3 h after mycolactone treatment, coinciding with ATF4 induction, and followed shortly after by CHOP. Indeed, this response to mycolactone is conserved widely between cell types (Fig. S2), although, as with other mycolactone-dependent responses, sensitivity varies^1,4,24–27^. Taken together, these data validate the translational profiling findings and suggest mycolactone may elicit a prototypical stress response.

### The mycolactone response involves multiple eIF2α kinases but not ER stress

**Fig. 2:**
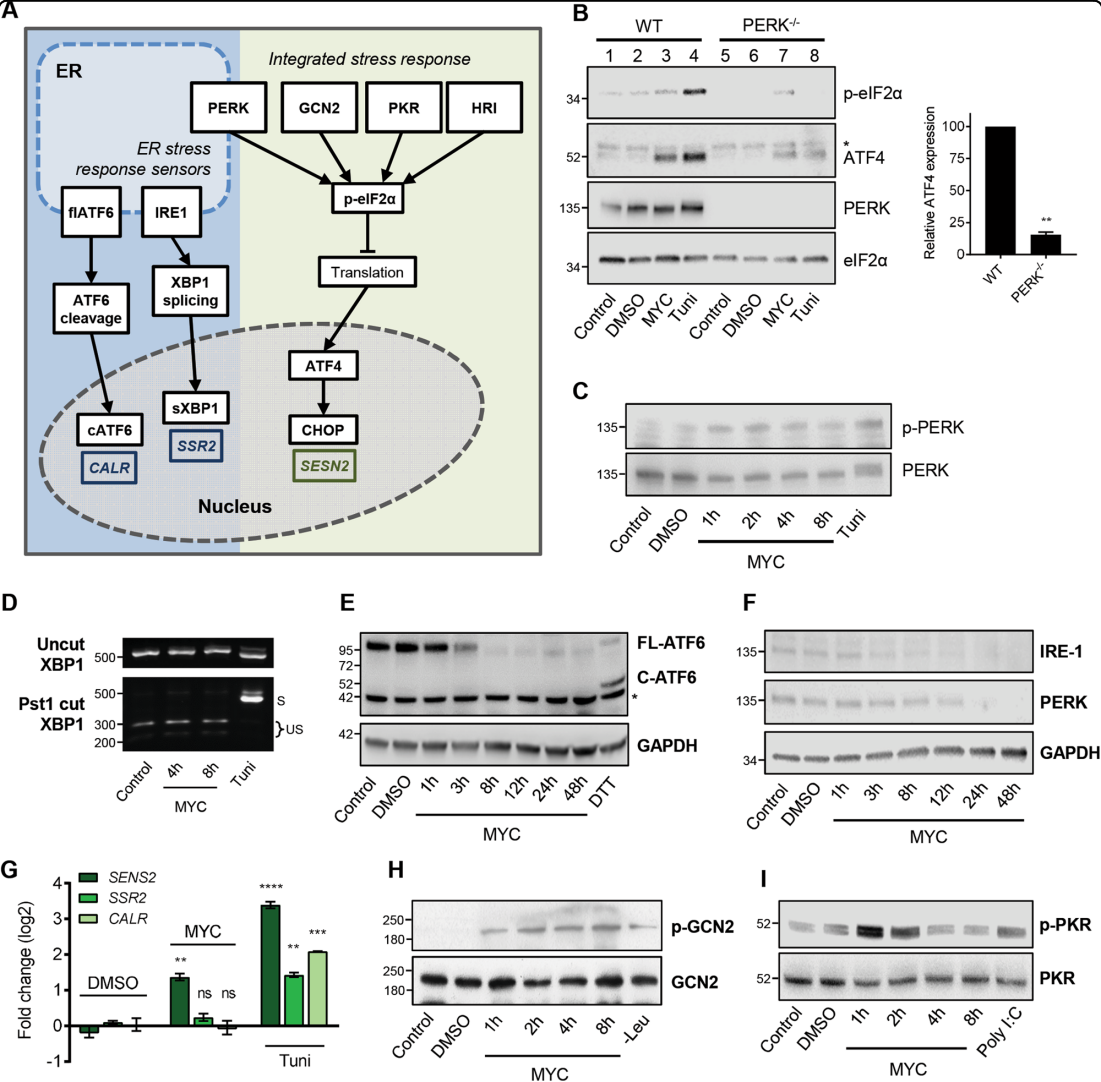
Mycolactone uncouples the integrated response from the unfolded protein response via a pathway that implicates multiple eIF2α kinases. **a** Cartoon representation of the ISR and ER stress response sensors and consequences. Genes that are specifically induced by the three ER sensors are shown. **b** Wild-type (WT) or knockout MEFs were treated with either mycolactone (MYC), DMSO or tunicamycin (Tuni), and the lysates were analysed by immunoblotting. * indicates a cross-reactive band. Relative semi-quantified signal intensities for ATF4 (Mean ± SEM, *n* = 3 independent experiments). **c**,** h**,** i** HeLa cells were treated as shown and lysates were analysed by immunoblotting. Representative data from *n* > 3 independent experiments. **d** HeLa cells were treated as shown. Total RNA was isolated and used as a template for RT-PCR of *XBP-1* (upper panel) which was then digested with *Pst1* and separated on a 2% agarose gel (lower panel). The migration of molecular weight markers in bp is indicated. S spliced, US unspliced. **e**,** f** HeLa cells were treated with DMSO for 48 h, DTT for 1 h or mycolactone (MYC) for the indicated duration (up to 48 h). Equal protein quantities in lysates were analysed by immunoblotting. * indicates a cross-reactive band. **g** HeLa cells were treated as shown for 10 h. Relative fold change (ΔΔCt) for steady-state mRNA levels determined by one-step qRT-PCR on total RNA (Mean ± SEM of three independent experiments). All immunoblots show the approximate migration of molecular weight markers in kDa. See also Fig. S3

The ISR overlaps with ER stress due to the participation of PERK in both pathways (Fig. 2a). Since the cellular target of mycolactone, the Sec61 translocon, is located in the ER membrane, we reasoned ER stress was a likely mediator. Mouse embryonic fibroblast MEFs respond rapidly to mycolactone, with ATF4 detected after 2 h exposure (Fig. S2B). As previously reported^12^, MEFs with a homozygous genetic deletion of PERK showed a strongly attenuated response to tunicamycin (Fig. 2b, lanes 4 and 8). In PERK^−^^/−^ MEFs, mycolactone-induced p-eIF2α was reduced compared to wild type (Fig. 2b, compare lanes 3 and 7), and ATF4 expression decreased by 85 ± 2.2% (*p = *0.0002). Furthermore, we detected modest, but rapid, phosphorylation of PERK in mycolactone-treated cells (Fig. 2c).

The three ER stress sensors (Fig. 2a) are thought to respond in parallel^17^; however, we have strong evidence that mycolactone activates PERK without discernible activation of IRE1 or ATF6. In accordance with previous findings^4^, IRE1 activation (identified by splicing of XBP1) was undetectable in mycolactone-treated cells (Fig. 2d) even at extended timepoints (Fig. S3A). Furthermore, no ATF6 cleavage was observed following mycolactone exposure (Fig. 2e and Fig. S3B). On the contrary, mycolactone caused depletion of full length ATF6, presumably because it is a Sec61-dependent type II TMP^6^. IRE1 and PERK are also depleted by mycolactone, but after extended exposure times (Fig. 2f). Induction of discriminatory target gene mRNAs downstream of the three sensors^28^ confirmed these findings. While tunicamycin upregulates all three gene, (Fig. 2g), mycolactone only induced Sestrin-2 (*SESN2*, downstream of the ISR, *p *= 0.0045) but not Signal Sequence Receptor Subunit 2 (*SSR2*, downstream of IRE1 and XBP1) or Calreticulin (*CALR*, downstream of ATF6).

Since these findings suggest that the stress induced by mycolactone is not being sensed in the ER, we asked whether other eIF2α kinases may also be involved in the response. We found a broad and rapid activation of eIF2α kinases (Figs. S3C, D). Phosphorylation of the nutrient sensor GCN2^29^ was evident after 1 h of exposure (Fig. 2h) and continued to increase up to 8 h. Transient phosphorylation of PKR (Fig. 2i), which normally senses viral dsRNA^11^ but also hyperosmotic stress in an RNA-independent manner^30^, may explain the residual ATF4 induction we observed in PERK^−/−^ GCN2^−/−^ MEFs (Fig. S3D). Hence, our results demonstrate that multiple eIF2a kinases are involved in the ISR in mycolactone-exposed cells.

### eIF2α phosphorylation and translational attenuation is required for ATF4 induction by mycolactone

**Fig. 3:**
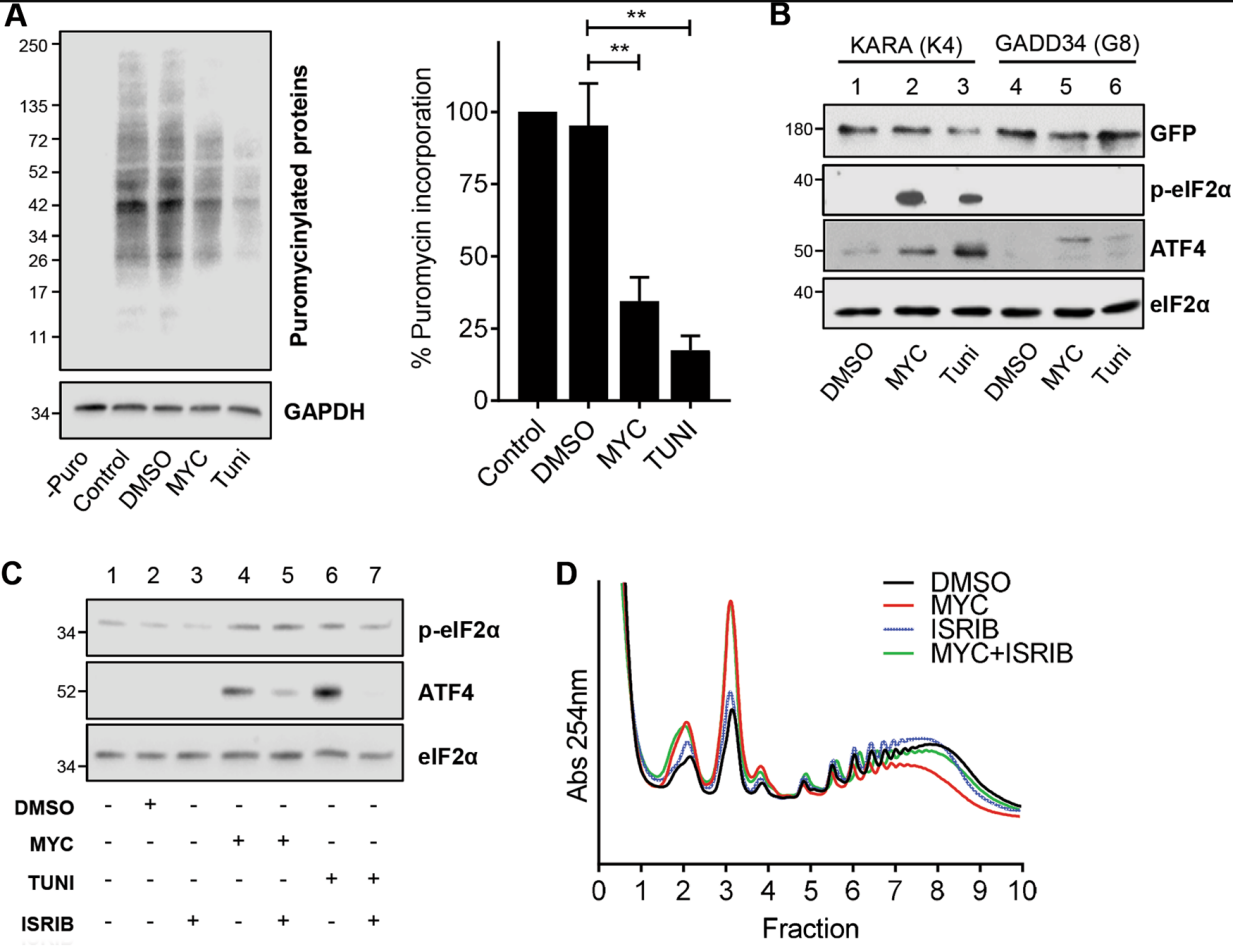
eIF2α phosphorylation and translational control drive ATF4 expression in cells exposed to mycolactone. **a** Immunoblot analysis of newly synthesised puromycilated proteins prepared from HeLa cells exposed to DMSO, mycolactone (MYC) or tunicamycin (Tuni) for 12 h. Relative quantified signal intensities are shown (Mean ± SEM, *n* = 3 independent experiments). **b** HeLa-gs cells stably expressing GFP-GADD34 (clone 8) or GFP-KARA (clone 4), an inactive mutant of GADD34, were exposed for 5 h. Lysates were analysed by immunoblotting. **c** HeLa cells were pre-treated with ISRIB for 1 h, followed by exposure to mycolactone for 8 h. Lysates were analysed by immunoblotting. **d** RAW246.7 cells were exposed to either DMSO, mycolactone or 100 nM ISRIB for 5 h. For co-incubation cells were pre-treated with ISRIB for 1 h prior to addition of mycolactone. Cell lysates were subject to polysome profiling. All immunoblots show the approximate migration of molecular weight markers in kDa. All data representative of at least three independent experiments

To confirm mycolactone induces the ISR, we used several different experimental approaches. First, we measured the impact of mycolactone on translation, observing a large reduction (66.5 ± 8.2% (*p *= 0.0056)) in puromycin incorporation (Fig. 3a). This finding, combined with the reduction in polysomes upon mycolactone treatment (Fig. 1), is consistent with reduced availability of the ternary complex expected following eIF2α phosphorylation. Second, we assessed the effect of mycolactone in cells stably overexpressing the p-eIF2α phosphatase GADD4. Here, mycolactone induced neither p-eIF2α nor ATF4 expression (Fig. 3b, lane 5). In control cells expressing KARA (a GADD34 (Val225Ala, Phe558Ala) variant unable to recruit PP1^14^), both ISR markers were detected after mycolactone treatment (Fig. 3b, lane 2). Third, pharmacological inhibition of the ISR was achieved with ISRIB, which activates the guanine nucleotide exchange activity of eIF2B, partially restoring eIF2-GTP levels and translation even during stress and despite high p-eIF2α levels^31,32^. ISRIB reduced the degree of ATF4 induction by mycolactone (Fig. 3c, compare lanes 4 and 5) and also partially reversed the mycolactone-dependent changes to polysome profiles (Fig. 3d). Taken together, these findings strongly support a model whereby mycolactone stimulates p-eIF2α, attenuating translation and driving ATF4 expression.

### The ISR initially protects against cell death by inducing autophagic responses

**Fig. 4:**
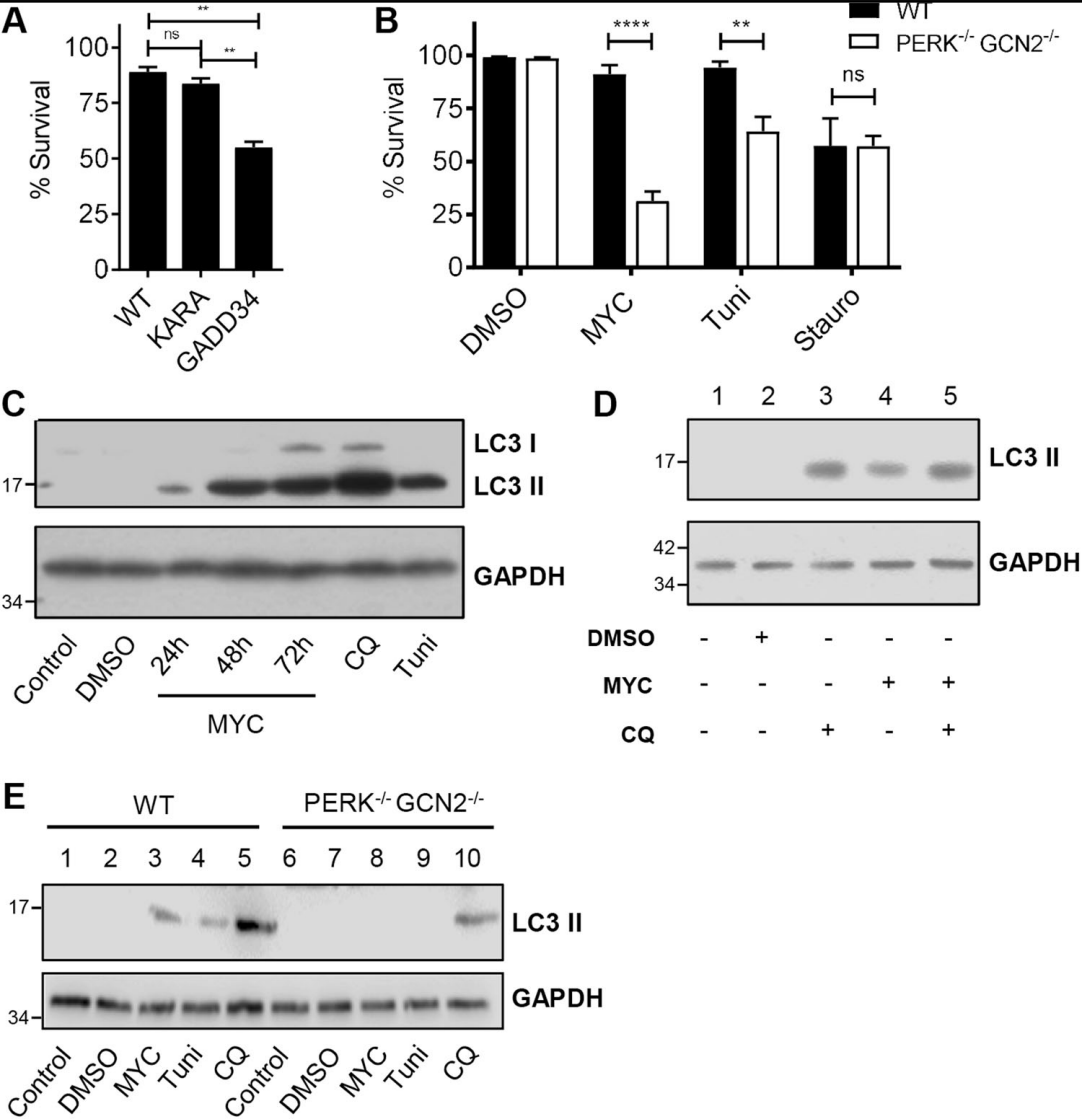
The phosphorylation of eIF2α protects cells by a mechanism that involves adaptive autophagy. **a** HeLa-gs cells stably expressing GFP-GADD34 (clone 8) or GFP-KARA (clone 4), an inactive mutant of GADD34, were treated with mycolactone for 4 days The number of apoptotic cells (positive for both active caspase 3/7 and PI) were determined for three fields and expressed as a proportion of total cells (Mean ± SEM *n* = 4 independent experiments). **b** Wild-type and PERK^−/−^ GCN2^−/−^ MEFs were treated with mycolactone for 24 h and analysed by confocal microscopy as in (**a**). **c**,** d** HeLa cells were treated as shown or with chloroquine (CQ) for 12 h. Equal protein quantities in lysates were analysed by immunoblotting. **e** WT and PERK^−^^/^^−^ GCN2^−/−^ MEFs were treated as in (**c**). Lysates were analysed by immunoblotting. All data representative of at least three independent experiments. All immunoblots show the approximate migration of molecular weight markers in kDa. See also Fig. S4

To determine the consequences of ISR activation, we compared survival of cells stably expressing GADD34 to wild-type and KARA cells. Cells exposed to mycolactone can persist for several days before succumbing to apoptosis^33–35^, although different cells show distinct kinetics (Fig. S4A). Here we followed cell death, as determined by activation of caspase 3/7 and PI staining, using time-points and concentrations where WT cells show minimal change. The inability to induce an ISR provided a significant loss of protection from mycolactone-induced cell death (55 vs. 89% surviving cells, *p *= 0.0042, Fig. 4a). Likewise, PERK^−/−^ GCN2^−/−^ MEFs also showed reduced survival (31 vs. 91%, *p *< 0.0001, Fig. 4b). Both cell types were equally susceptible to the control staurosporine.

This altered susceptibility can be explained, at least in part, by an inability to induce autophagy^28^. Others have previously shown that mycolactone induces this adaptation to stress^34^. However, whereas Gama et al. ascribed autophagy induction to cytoskeletal changes, we show it is linked to the mycolactone-dependent ISR. We probed processing of microtubule-associated protein 1 light chain 3 (LC3-I), through lipidation, to LC3-II. Mycolactone, like tunicamycin and chloroquine, causes accumulation of LC3-II in HeLa cells (Fig. 4c) and other cell types (Fig S4B). Co-incubation of cells with mycolactone and chloroquine (an inhibitor of autolysosome degradation^36^) further increased levels of LC3-II (Fig. 4d, lanes 4 and 5), thus ruling out reduced autophagosome turnover as the cause of LC3-II accumulation. That autophagy is induced in a stress-dependent manner similarly to tunicamycin^37^ is supported by experiments in PERK^−/−^ GCN2^−/−^ MEFs, which retained chloroquine-mediated but not mycolactone-induced induction of LC3-II (Fig. 4e, lanes 3 and 8).

### Chronic exposure to mycolactone causes death via the ATF4/Bcl-2/Bim route

**Fig. 5:**
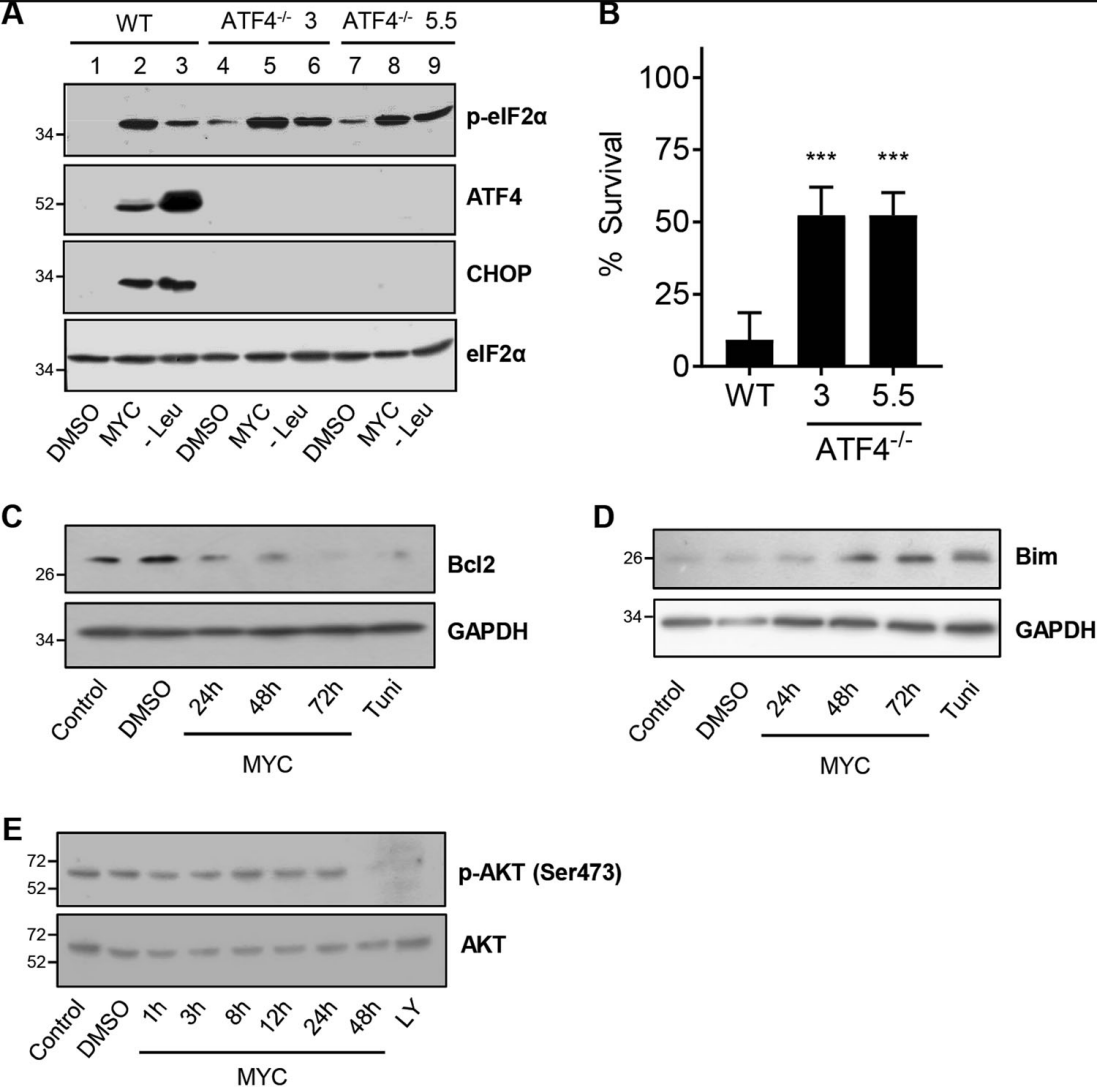
ATF4 promotes mycolactone-mediated cytotoxicity. **a**,** b** Wild-type HeLa cells and two different ATF4^−/−^ clones (3 and 5.5) were treated with either DMSO, mycolactone (MYC) or starved of leucine (Leu^−^). **a** Lysates from 24-h-treated cells were analysed by immunoblotting. **b** After 4 days the % survival of cells was determined by staining of cells with propidium iodide (PI), cell event (detects active caspase 3/7) and DRAQ5. The number of live cells (negative for both active caspase 3/7 and PI) in three fields was determined and expressed as a proportion of total cells (Mean ± SEM, *n* = 3 independent experiments). **c**−**e** HeLa cells were treated as shown or with LY294002 (LY) for 1 h. Equal protein quantities in lysates were analysed by immunoblotting. All immunoblots show the approximate migration of molecular weight markers in kDa. See also Fig. S5

ER stress responses, while initially protective, can also be damaging to cells, particularly in the face of chronic stress. These latter effects are driven not only by ATF4/CHOP but also IRE1^38^. Investigating the contributions of the different pathways normally requires genomic approaches as there are no known examples of inhibitors that activate PERK without also activating the other two ER sensors and CHOP is also a transcriptional target of both XBP-1 (IRE1) and ATF6^38,39^. We are now able to shed light on this by investigating the effects of mycolactone via the ISR in cells with and without ATF4 (Fig. S5). In two independent CRISPR/Cas9 generated knockout clones, neither ATF4 nor CHOP were induced by mycolactone or Leucine starvation, despite normal levels of p-eIF2α (Fig. 5a, compare lane 2 with lanes 5 and 8), providing further evidence of UPR absence in mycolactone-treated cells. Both ATF4 knockouts have a slightly elevated endogenous level of p-eIF2α (Fig. 5a, compare lane 1 with lanes 4 and 7), likely due to lowered expression of expression of GADD34, a downstream target of ATF4^40^.

ATF4 knockout clones were protected from mycolactone-mediated cell death (9 vs. 52% cells surviving, *p *= 0.0004, Fig. 5b), likely due to the absence of CHOP induction, which is known to promote cell death by altering expression of the pro-apoptotic Bim and pro-survival Bcl-2^41^. In WT cells, mycolactone exposure decreased Bcl-2 expression from 24 h (Fig. 5c) and increased Bim from 48 h (Fig. 5d) suggesting that ISR-dependent induction of ATF4 and CHOP causes a shift in the pro/anti-apoptotic mechanisms to favour apoptosis from 48 h onward. Although upregulation of Bim by mycolactone has been ascribed to direct inhibition of mTORC2 signalling^10^, given the known cross-talk between eIF2α and mTORC2 pathways^42,43^ it is also possible that loss of mTORC2-dependent Akt phosphorylation at Ser473 between 24 and 48 h after exposure (Fig. 5e) is secondary to induction of ATF4.

### ATF4 induction depends on Sec61 blockade by mycolactone

**Fig. 6:**
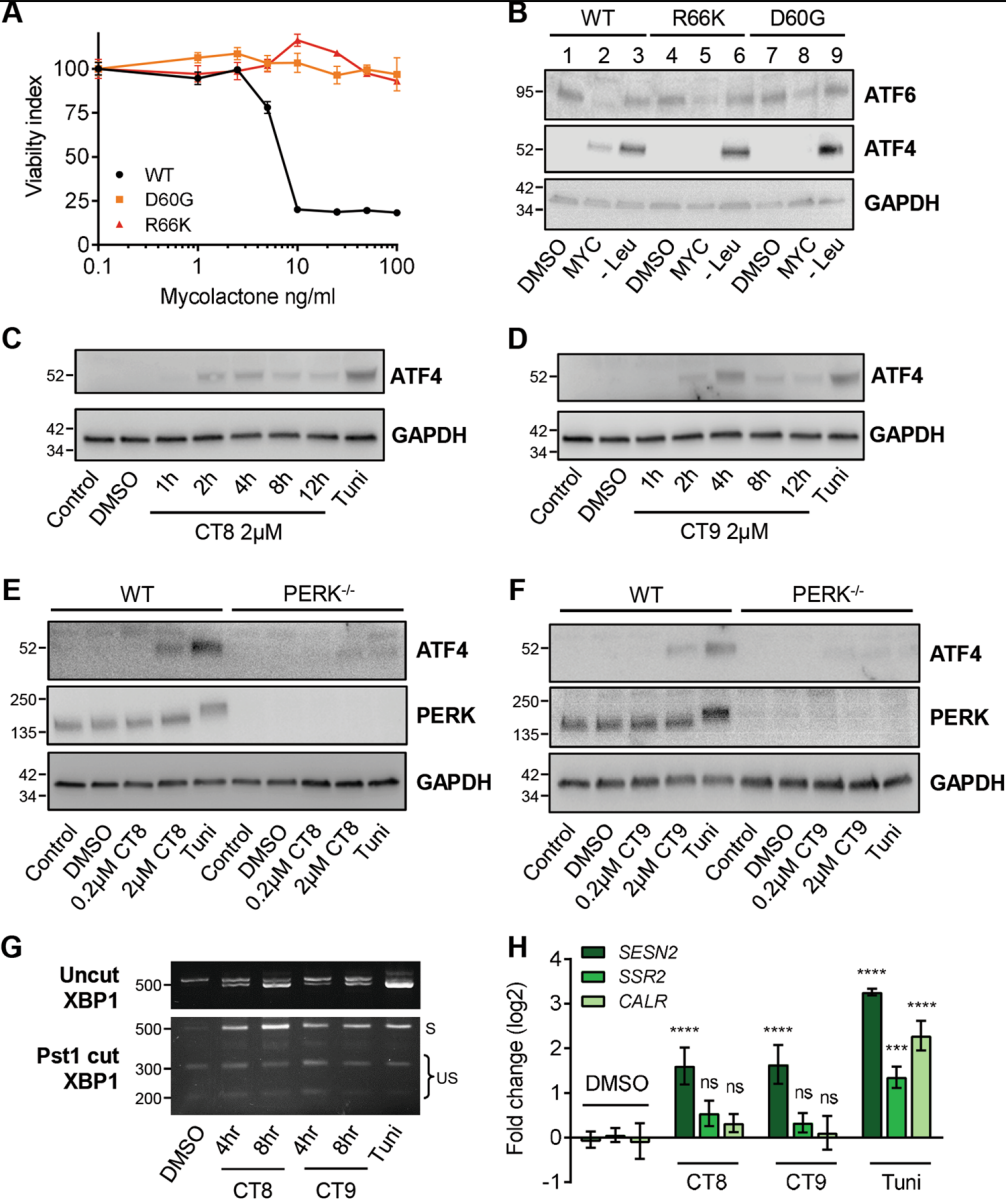
Uncoupling of the ISR from ER stress is a consequence of mycolactone’s effect on the Sec61 translocon, but is not a general feature of translocation inhibition. **a**,** b** An unbiased screen for mycolactone-resistant clones was performed in HCT-116 cells, yielding clones with heterozygous missense mutations in *Sec61A1*. Parental HCT-116 cells and representative clones with D60G and R66K were analysed. **a** Normalised viability index of cells treated with mycolactone for 5 days, assessed by MTT assay. **b** Immunoblot of lysates that were treated with DMSO, mycolactone or starved of Leucine (Leu^−^) for 24 h. **c**,** d** Wild-type MEFs were treated with CT8 (**c**) or CT9 (**d**) for the indicated times or tunicamycin (Tuni). Lysates were analysed by immunoblotting. **e**,** f** Wild-type (WT) or PERK^−/−^ MEFs were treated as shown and the lysates were analysed by immunoblotting. **g** Wild-type MEFs were treated as shown. Total RNA was isolated and used as a template for RT-PCR of *XBP-1* (upper panel) which was then digested with *Pst1* and separated on a 2% agarose gel (lower panel). The migration of molecular weight markers in bp is indicated. S spliced, US unspliced. All data representative of at least two independent experiments. **h** HeLa cells were treated as shown for 10 h. Relative fold change (ΔΔCt) for steady-state mRNA levels (Mean ± SEM, *n* = 3 independent experiments). All immunoblots show the approximate migration of molecular weight markers in kDa

To verify that mycolactone responses arise from Sec61 inhibition, we used random mutagenesis with ENS^44^ to generate mycolactone-resistant cells in the DNA repair-defective cell line HCT-116. Eight of nine independent, mycolactone-resistant clones analysed to date have one of two heterozygous mutations in the *SEC61A1* gene locus (Asp60Gly and Arg66Lys). These cells are highly resistant to the cytotoxic effects of mycolactone (Fig. 6a) and show reduced depletion of ATF6, used here as a surrogate for translocation blockade (Fig. 6b). In line with heterozygosity, restoration of ATF6 levels is only partial. As expected, all cells induced ATF4 in response to leucine starvation (Fig. 6b). Remarkably, and in contrast to parental cells, no ATF4 was induced in Sec61-mutant cells exposed to mycolactone (Fig. 6b) strongly suggesting ATF4 induction is associated with the ability of mycolactone to alter translocon functionality.

In an alternative approach, we compared the mycolactone response with that of two variants of cotransin. CT8 is a selective inhibitor of translocation, whereas CT9 has broad-spectrum effects^9,45^. Both drugs induce ATF4 expression at higher doses (2 µM), albeit with slightly different kinetics (Fig. 6c, d; and cf. Fig. S2B), and less strongly than mycolactone. A weak ATF4 signal could be detected in cotransin-treated PERK^−/−^ MEFs (Fig. 6e, f), suggesting other kinases may also participate in the response to cotransins. Nevertheless, an uncoupling of the ISR from ER stress does not appear to be a common feature of translocation inhibition, since partial XBP1 splicing was also detected (Fig. 6g), coinciding with a small induction of *SSR2* expression, though not statistically significant (Fig. 6h).

## Discussion

We have identified an unusual property of the *M. ulcerans* exotoxin and Sec61-dependent translocation inhibitor, mycolactone, which is characterised by activation of PERK and other eIF2α kinases without concomitant activation of IRE1 and ATF6. This study therefore not only sheds light on the molecular mechanisms driving cell death in BU disease but also reveals highly selective cross-talk between the ER and the cytosol during stress.

The uncoupling of PERK activation from the other ER stress pathways is remarkable because stressful stimuli such as tunicamycin^41^ usually activate the ISR and ER stress responses in unison, since loss of  BiP binding activates each pathway^46^. Furthermore, in addition to PERK, at least two other eIF2α kinases contribute to the cellular response to mycolactone (PKR and GCN2) and no single kinase is solely responsible for the subsequent eIF2α phosphorylation and ATF4 induction. Such multiple-activation of eIF2α kinases has previously been shown to occur following UVB irradiation and in conditions causing oxidative stress (e.g. arsenite or H_2_O_2_)^47^, although here the UPR is also activated. Mycolactone has previously been shown to cause ROS production^48^, and inhibition of this by the addition of a combination of antioxidants (deferoxamine and TEMPOL) reduced cytotoxicity. Since selective activation of the ISR is dependent on mycolactone’s actions at the Sec61 translocon, future experiments will examine whether oxidative stress or production of other metabolites triggered by the inhibition of translocation might drive the response.

One explanation for the lack of UPR in mycolactone-exposed cells is the Sec61-depedence of the ER stress sensors themselves. ATF6 is a type II TMP, a class of protein that is particularly susceptible to mycolactone inhibition^5^. Inhibition of translocation leads to cytosolic degradation of newly synthesised proteins^4^. Since the half-life of ATF6 is ~2 h^49^ the observed decrease in cellular levels is not unexpected and could explain the failure to trigger this arm of the UPR. However, IRE1 and PERK are type I membrane proteins, which show a more variable response to mycolactone in vitro^5^ and have longer half-lives^46,50^. Since IRE1 is still detectable ~12 h after mycolactone addition, the lack of XBP1 splicing or SSR2 induction is less readily explained.

Taken together, our findings support a model in which mycolactone-mediated stress at the ER interface can be sensed predominantly in the cytosol, but responses are not contained within a single compartment. This supports recent findings of important cross-talk between the ER and the cytosol^51^. Others have previously examined the impact of translocation blockade on similar pathways. Notably, both genetic knockdown of translocon components^28^ or chemical treatment with other translocation inhibitors ESI^52^, CAM741^53^ or cotransins (this manuscript), all cause activation of IRE1. Thus, the uncoupled response to mycolactone is not common to translocation inhibition per se. The differences between mycolactone and cotransin are unexpected because of the overlap in missense mutations conferring resistance^9,44,45^. Furthermore, mycolactone competes for cotransin binding to Sec61α implying a coincident binding site^9^. Nevertheless, the downstream effects seem different, suggesting distinct mechanisms of action. As cotransin is a cyclodepsipeptide and mycolactone is a polyketide lactone^8^, with very different chemical properties, they may interact with the translocon in distinct ways, even if the binding site is similar. Mycolactone, as the more potent agent, may also lead to a faster loss of function within the ER.

A large body of research supports the contention that the canonical ISR, by decreasing translation and activating cytoprotective processes such as autophagy, is initially protective. For instance, PERK activation can induce autophagy and several autophagy genes are downstream of ATF4^54^. Conversely, activation of the ISR has also been shown to promote cell death. The nature of the response obtained in practice is thought to be a function of both extent and duration of the stress. For cells exposed to environmental stress, ISR activation can be at first beneficial; however, in the latter stages of the response, ATF4 and CHOP restore protein synthesis by recruiting their downstream target GADD34 to de-phosphorylate eIF2α, leading to oxidative stress and cell death^40^. In addition, CHOP also promotes cell death by altering the expression of apoptosis regulators such as Bim, Bcl2, Bax and Bad^41^.

Two different genetic models with reduced ability to phosphorylate eIF2α die at an accelerated rate following mycolactone exposure, suggesting the mycolactone-induced ISR operates by a similar mechanism. Consistent with this, the activation of the cytoprotective autophagic response by mycolactone was also lost in PERK^−/−^GCN2^−/−^ cells. With prolonged exposure to mycolactone, there was a shift towards a pro-apoptotic response, with increased expression of pro-apoptotic proteins. Conversely, cells unable to make ATF4 were more resistant to the toxic effects of mycolactone indicating that this is a key determinant of the fate of cells. However, although cell death was delayed, it was not completely prevented, suggesting additional triggers of apoptosis in cells exposed to mycolactone, or other pathways that manifest in the absence of ATF4.

In this regard, there remain additional candidates identified in the translational profiling that require investigation; thus the full extent of mycolactone-responsive genes and pathways awaits discovery. Given the single 5 h time point used, this data set likely includes both primary targets (like ATF4) and secondary response genes. For instance, reduced polysomal association of several interferon response genes (*IFIT1*, *IFIT3* and *IGTP*) could be explained by the profound inhibition of type I interferon  production by these cells^4,47^. However, it is important to recognise that enhanced translation does not necessarily correlate with increased protein expression, should the translated transcripts be subject to translocation inhibition and thus premature degradation^4^. Nevertheless, it would be interesting to compare the transcriptional and translational effects of mycolactone in mutant lines lacking any or all of the eIF2α kinases, or the ATF4^−/−^ cells, to further dissect the pathway.

Mycolactone seems unique in its ability to induce stress in the cytosol despite an initial priming event at the ER membrane. The global effect of mycolactone is widely conserved between varied cell types derived from both humans and mice (for instance, refs.^4,9,35^). Our discovery has implications for treatment of *M. ulcerans* infection. BU lesions are characterised by widespread tissue damage and cell death, and wound healing even after antibiotic treatment is extremely slow and the presence of residual mycolactone could be a contributory factor. Prolonged exposure to stress leads to the activation of pathways that have been shown to drive *M. ulcerans*-infection-induced apoptosis^10^. Could this pathway therefore be targeted to treat BU? The answer is not straightforward, since reduced p-eIF2α enhanced rather than diminished susceptibility to mycolactone. In other diseases, such as dementia, reducing the ISR only appears to be protective at early stages of the disease^55,56^. Unravelling the mechanisms of mycolactone activity is valuable for understanding the pathogenesis of BU but additionally provides insights useful in the development of chemotherapeutic agents that modulate the ISR and ER stress response.

## Material and methods

### Reagents

We used synthetic mycolactone A/B (kind gift from Prof. Yoshito Kishi, Harvard University) throughout these investigations. Unless specified, all other reagents are from Sigma-Aldrich.

### Cell culture and treatment

PERK^−/−^, GCN2^−/−^, PERK^−/−^ GCN2^−/−^ MEFs or their wild-type counterparts have been described previously^57,58^, while all other cells were obtained from ATCC. To allow for stable overexpression of the KARA and GADD34 clones (see below), specific-geneticin-sensitive HeLa cells were used, referred to here as HeLa-gs. All cells were maintained in high-glucose DMEM supplemented with 10% FBS at 37 °C and 5% CO_2_ and in the case of PERK^−/−^, GCN2^−/−^, PERK^−/−^ GCN2^−/−^ MEFs and ATF4^−/−^ HeLa cells, 1 mM non-essential amino acids, 50 µM β-mercaptoethanol, 100 units/ml penicillin and 100 μg/ml streptomycin were also added. Unless otherwise noted, mycolactone was used at 31.3 ng/ml and the vehicle control was DMSO diluted to the same extent (0.025%). Initial experiments with RAW264.7 cells utilised mycolactone at 125 ng/ml as previously described^4^. Various inducers of the integrated stress response were utilised. Induction of ER stress routinely achieved by adding 5 µg/ml tunicamycin to the medium followed by incubation for up to 12 h. To activate GCN2, cells were grown in leucine-free DMEM/Nutrient Mixture F-12 Ham for up to 8 h while PKR activation was achieved by transfecting cells with 10 µg/ml Poly I:C for 4 h. Other inducers used were cycloheximide (10 µg/ml), chloroquine (50 µM), DL-Dithiothreitol (1 mM), staurosporin (Calbiochem, 1 µM), Cotransin-8 (CT8; 2 µM), Cotransin-9 (CT9; 2 µM)^45^ and ISRIB (100 nM).

### Polysome profiling, RNA isolation and analysis

Polysome profiling was carried out as previously described^4^. Briefly, cells were incubated with 10 μg/ml cycloheximide (CHX) for 10 min at 37 °C and 5% CO_2_ and harvested by scraping into PBS/CHX and spun at 450 × *g* for 5 min at 4 °C. The cell pellet was resuspended in 500 μl lysis buffer (15 mM TrisCl pH 7.5, 300 mM sodium chloride, 15 mM magnesium chloride, 10 mg/ml heparin, 100 μg/ml CHX and 1% (v/v) Triton-X-100). Lysates were clarified by centrifugation at 21,000 × *g* for 1 min at 4 °C and supernatants snap frozen in liquid nitrogen. To separate polysomes, samples were layered onto a 10–50% sucrose gradient in lysis buffer and centrifuged in a SW40Ti rotor (Beckmann Coulter) at 38,000 rpm for 2 h. Gradients were fractionated using a FoxyR1 collection system (Teledyne ISCO) and RNA was extracted from fractions as previously described^4,59^. For northern blotting, extracted RNA was separated on 1% agarose/formaldehyde gels and transferred onto Hybond N+ membranes. Blots were probed in Ultrahyb solution (GE Healthcare) with ^32^P-labelled cDNA probes washed and exposed to a phosphorimager screen (Bio-Rad). Full length coding-region cDNA probe for actin was already available^22^. Murine ATF4 probe was prepared by amplification and cloning of full-length coding-region cDNAs from RAW264.7 cells exposed to mycolactone.

### Microarray analysis

RAW297.4 cells (1–2×10^7^) were incubated with or without 125 ng/ml mycolactone for 1 h, then stimulated with 100 ng/ml TLR-grade LPS (Enzo Life Sciences) for 4 h. Cells were treated with 10 µg/ml CHX before harvesting for polysome isolation. Polysomes were prepared and RNA purified as described in Hall et al.^4^. Subpolysomal (fractions 1–5) and polysomal fractions (fractions 6–10) were pooled and precipitated twice with lithium chloride, then purified through RNA Clean and Concentrate-5 columns (Zymo Research). RNA quality was confirmed by Bioanalyser (Bio-Rad). Cy5 and Cy3 labelled cRNA with Spike-in was prepared from pooled RNA (~150 ng each) using a Two Colour Low Input Quick Amp Labelling Kit (Agilent). Spike-in concentration was adjusted according to sample RNA concentration. Polysomal and sub-polysomal cRNAs (825 ng each) were pooled and used to probe Agilent Mouse Gene Expression v2 4 × 44 K slides according to the manufacturer’s instructions. Dye swaps were carried out to compensate for dye bias. Fluorescence intensity was measured with an Agilent Microarray Scanner. Data was analysed by the method described in ref.^23^, with some variations. Briefly, background was corrected by the normexp method. Low intensity values (<2 SD above background) were filtered out. Normalisation was carried out within arrays by the Loess method and between arrays by the Scale method. After dye-swap correction, the fold change in polysomal association for individual transcripts due to mycolactone was calculated from the normalised data using the following formula:

$${{\rm FC}} = \frac{{{{\rm mval}}\,({\rm{Mycolactone}}\,+\,{\rm{LPS}})}}{{{\rm{mval}}\,{\rm{(LPS)}}}},$$ where $${\rm{mval}} = \frac{{{\rm{mean}}\,{\rm{values}}\,{\rm{of}}\,{\rm{polysomal}}\,{\rm{fractions}}\,{{(n = 3)}}}}{{{\rm{mean}}\,{\rm{values}}\,{\rm{of}}\,{\rm{subpolysomal}}\,{\rm{fractions}}\,{{(n = 3)}}}}.$$

Transcripts showing a significant change in translational efficiency after exposure mycolactone were identified by Rank Product analysis of the entire data set of three biological repeats. This rank product analysis rules out changes in transcription that may occur and, while it yields a lower number of positive hits than other approaches, it leads to a higher validation rate for translationally regulated targets^23^. Gene transcripts that were enriched in the polysomal or sub-polysomal pools (reflecting enhanced and reduced translation, respectively) were subject to PANTHER Overrepresentation Test (release 20160715) to identify significantly overrepresented gene ontology (GO) groups (*p* < 0.05).

### Preparation of protein lysates and immunoblot analyses

For western blots, cells were lysed either in RIPA buffer (Sigma) supplemented with protease inhibitors (Sigma) and phosphatase inhibitors (1 mM sodium pyrophosphate, 1 mM PMSF, 5 mM sodium fluoride and 1.75 mM β-glycerophosphate) or gel sample buffer followed by sonication for 30 s. Following RIPA lysis, lysates were clarified by centrifugation, and the protein content was determined using Pierce BCA protein assay kit (Thermo Fisher) according to the manufacturer’s recommendation. For immunoblots, equal amounts of each protein sample were separated in an SDS-polyacrylamide gel followed by conventional blotting and a broad range polypeptide marker (Thermo Fisher) was used to determine the molecular weight of proteins. The antibodies used in this study were as follows: anti-p-PERK, p-PKR and PKR (Santa Cruz Biotechnology); anti-p-eIF2α (Ser-51) and anti-GFP (Invitrogen); anti-Bcl-2, Bim and LC3B (ProSci); anti-p-GCN2 (Thr-899) (Abcam); and anti-GAPDH (Ambion). With the exception of the ATF6 antibody that has been described previously^17^, all other antibodies were purchased from Cell Signaling Technology and the HRP-conjugated secondary antibodies were from Life Technologies. The size of each protein was determined by comparisons to molecular weight markers. CHOP (∼27 kDa), GAPDH (∼37 kDa), ATF4 (49 kDa), p-eIF2α/eIF2α (∼38 kDa), GFP GADD34/KARA (∼180 kDa), ATF6 (full-length ∼90 kDa, cleaved ∼50 kDa), IRE1 (∼130 kDa), p-GCN2/GCN2 (∼220 kDa), p-PERK/PERK (∼140 kDa), p-PKR/PKR (∼50 kDa), GAPDH (∼37 kDa), LC3B II (∼17 kDa), Bcl2 (∼28 kDa), Bim (∼23 kDa), p/AKT/AKT (∼60 kDa). All immunoblotting results are representative of at least three independent experiments. Where quantitation was performed, pixel density was assessed using ImageJ analysis of non-saturated images and data were normalised to an appropriate loading control (eIF2α or GAPDH).

### PCR and qPCR

Total RNA was extracted using a Qiagen RNAeasy kit. Off-the-shelf gene expression assays used were: GAPDH, 4352934; ATF4, Mm_00515324_m1; CHOP, Mm_01135937_g1; SESN2, Hs_00189032_m1; CALR, Hs_00162346_m1 and SSR2, Hs_0023012_m1. Real-time one-step qRT-PCR was carried out with either One-Step RT-PCR Master Mix (Applied Biosystems) on Quantstudio 7 flex real-time PCR system (Applied Biosystems). Primers used for RT-PCR analysis of XBP1 mRNA splicing by IRE1 were 5′-AAACAGAGTAGCAGCTCAGACTGC-3′ and 5′-TCCTTCTGGGTAGACCTCTGGGAG-3′.

### Puromycin labelling

Cells were incubated in complete medium containing 9 µM puromycin and 91 µM emetine at 37 °C for the last 5 min of a 12 h exposure before harvest. Cells were lysed with gel sample buffer pre-heated to 95 °C after which the lysate was boiled for a further 5 min and sonicated for 30 s. Immunoblotting was performed with anti-puromycin (Merck) and scanned images were quantified using ImageJ software.

### Transfection

HeLa cells were either transiently or stably transfected using Lipofectamine LTX (Invitrogen). Reverse transfection of 5×10^4^ cells was routinely carried out on a 24-well tissue culture plate using 300 ng plasmid DNA and 1 µl Lipofectamine LTX according to the manufacturer’s recommendation. EGFP-C1 GADD34 and EGFP-C1 KARA plasmids were kindly provided by Shirish Shenolikar (Duke University) while two plasmids each encoding a Cas9 nickase and an ATF4-specific guide RNA were obtained from Santa Cruz (sc-400155). Sixteen hours post-transfection, plasmid uptake was assessed by observing the cells under a fluorescent microscope for expression of GFP. For transient transfection with the ATF-4 double nickase plasmids, both mock and plasmid transfected cells were transferred into a 100 mm dish and selection was carried out with 200 ng/ml puromycin. Media was replaced every 2 days and 96 h post transfection, selection medium was replaced with DMEM supplemented with FBS, 1 mM non-essential amino acids and 50 µM β-mercaptoethanol (these growth conditions are specific to ATF4 knockout cells). For stable transfection with EGFP-C1 GADD34 and EGFP-C1 KARA plasmids, selection was initiated 48 h post transfection with DMEM containing 400 µg/ml G418. In both cases, when distinct colonies were observed, they were picked and moved to a 24-well plate followed by expansion and gene expression or deletion was confirmed by western blot analysis of cell lysates.

### Viability assays

Cells in a 96-well plate were treated as required and stained with 0.3 µg/ml of PI (BD Biosciences), 2% (v/v) CellEvent (Invitrogen) and 3 µM DRAQ5 (Biostatus) to label nuclei followed by incubation in the dark for 30 min. Stained cells were observed using a Nikon A1 confocal microscope. Active caspase-3/7, nuclear and PI staining of the same field were observed in three different fields representing the top, bottom and middle part of the plate. The number of active caspase 3/7 (green channel) and PI (red channel)-positive cells was counted for each field and expressed as a percentage of the total number of cells (based on DRAQ5 staining, blue channel) in the field.

### Unbiased screen for mycolactone-resistant clones using ENS mutagenesis

DNA damage repair-resistant Hct-116 cells were randomly mutagenised according to the method of Junne et al.^44^. Briefly, cells were treated with ethyl methane sulphonate at the IC_20_ for 1 h, reseeded at 15,000 cells/cm^2^ and allowed to recover overnight. Cells were then treated with 10 ng/ml mycolactone (the minimal inhibitory concentration for this cell line) every 5 days for 3 weeks after which colonies of mycolactone-resistant cells began to emerge. Colonies were picked and expanded in a 24-well plate that also contained 10 ng/ml mycolactone. No mycolactone-resistant clones were obtained from control cells that had not been mutagenised. To test each resistant clone, cells were further expanded before RNA extraction and parallel testing for mycolactone-sensitivity by IC_50_ at 5 days using an MTT assay^25^. In order to determine if mutations were present in SEC61A1, mRNA was reverse transcribed and the coding region amplified by PCR in four overlapping fragments (Sec61A1_frag1F; TAGCACTGACGTGTCTCTCG, Sec61A1_frag1R; TCCCCATACATCCCGGTCAT, Sec61A1_frag2F; CTTCAACGGAGCCCAAAAGT, Sec61A1_frag2R; GTGTTGTACTGGCCACGGTAG, Sec61A1_frag3F; TCATCGCCACCATCTTTGTCTT, Sec61A1_frag3R; GGACCATGGAGGTCTCTCGG, Sec61A1_frag4F; TATACATAGTGTTCATGCTGGGCT, Sec61A1_frag4R; ACACAGTGGAATGAAAGAATACGA) then subject to Sanger sequencing.

### Statistical analysis

Data were analysed using Graphpad Prism v.6 software. For semi-quantification of immunoblots normalised to control levels, a one sample *t*test was employed. For qRT-PCR, two-way ANOVA with Holm−Sidak test for multiple comparisons was performed on log_2_ transformed data. All other comparisons utilised either a one- or two-way ANOVA with Dunnet’s or Holm−Sidak test for multiple comparisons as appropriate. n.s., not significant, *p ≤ 0.05, **p ≤ 0.01, ***p ≤ 0.001, ****p ≤ 0.0001.

## Electronic supplementary material

Supplemental figure legends(PDF 183 kb)

Table S1(PDF 360 kb)

Table S2(PDF 401 kb)

Figure S1(PDF 220 kb)

Figure S2(PDF 714 kb)

Figure S3(PDF 179 kb)

Figure S4(PDF 45 kb)

Figure S5(PDF 15 kb)
