# Supplementary material for: Inhibition of Sec61-dependent translocation by mycolactone uncouples the integrated stress response from ER stress, driving cytotoxicity via translational activation of ATF4
Source: Cell Death Dis. 2018 Mar 14;9(3):397. doi: 10.1038/s41419-018-0427-y (PMC5852046; doi:10.1038/s41419-018-0427-y)
Supplement: Supplementary file 1 — Supplemental figure legends(PDF 183 kb) [file 41419_2018_427_MOESM1_ESM.pdf]

Supplemental figure legends

Figure S1. Polysome profiles of different cell treatments

(A) HeLa cells were treated with DMSO, 31.3 ng/ml mycolactone or tunicamycin for 16h

(B) RAW264.7 cells were treated with DMSO or tunicamycin (Tuni) for 5hrs

In both cases, cells were harvested and lysed in the presence of cycloheximide and polysomes were separated on a 10–50% sucrose gradient. RNA throughout the gradient was quantified by absorbance at 254 nm.

Figure S2. ATF4 and CHOP are induced upon mycolactone exposure in many different cell types

(A, B) Different types of cell lines and primary cells were treated with either 31.3 ng/ml mycolactone (MYC) for the indicated times or different doses of mycolactone for 12hrs. Tunicamycin treatment for 3hrs was the positive control. Lysates were analysed by immunoblotting

Figure S3. No evidence for IRE1 or ATF6 activation over a range of mycolactone exposure times, but the cytosolic stress is driven by multiple kinases

(A) HeLa cells were treated with DMSO, tunicamycin (Tuni) for 8hrs or mycolactone (MYC) for the indicated times. Total RNA was isolated and used as a template for RT-PCR of *XBP-1* (upper panel) which was then digested with *Pst1* and separated on a 2% agarose gel (lower panel). S; spliced, US; unspliced

(B) HeLa cells were treated with DMSO for 4hrs, DTT for 1hr, or mycolactone (MYC) for the indicated duration (up to 48 hrs). Equal protein quantities in lysates were analysed by immunoblotting. * indicates a non-specific band.

(C & D) Wild type (WT) or the indicated knockout MEFs were treated with either mycolactone (MYC), DMSO, tunicamycin (Tuni) or starved of leucine (Leu-) for 2hrs, and the lysates were analysed by immunoblotting. In each case the right hand panel shows relative semi-quantified signal intensities for ATF4 compared to WT MEFs (Mean±SEM, n=3 independent experiments). One sample t-test * $P \leq 0.05$, ** $P \leq 0.01$.

Figure S4. Death kinetics and autophagy induction in different cells

(A) Wildtype HeLa and HeLa-gs cells and MEFs were treated with mycolactone for various times analysed by fluorescent microscopy following staining of cells with propidium iodide (PI), Cell event and DRAQ5. The number of apoptotic cells (positive for both active caspase 3/7 and PI) were determined for 3 fields and expressed as a proportion of total cells mean±SEM (n = 4). One way ANOVA with Dunnet's test for multiple comparisons test. * $P\leq$

0.05, *** $P \leq 0.001$, **** $P \leq 0.0001$. For HeLa gs cells, since these cells were expressing GFP-tagged KARA, only PI staining was used (n=1).

(B) MEFs were treated with either DMSO for 24hrs, mycolactone (MYC) for the indicated times, tunicamycin (Tuni) for 48hrs or chloroquine (CQ) for 12hrs. Equal protein quantities in lysates were analysed by immunoblotting

Figure S5. Confirmation of ATF4 knockout by CRISPR

(A) Genomic DNA was extracted from clones 3 and 5.5 and a portion of the ATF4 gene flanking the targeted region was amplified and visualised. M: marker.

(B) Sanger sequencing of clones 3 and 5.5 showing the targeted region of the ATF4 gene. gRNA targeting sites on the sense and antisense strand are depicted in blue and purple, respectively; PAM sequences are shown in red; mutated sequence is shown in green; INS: insertion, NT: nucleotide.
