# Supplementary material for: Inhibition of Sec61-dependent translocation by mycolactone uncouples the integrated stress response from ER stress, driving cytotoxicity via translational activation of ATF4
Source: Cell Death Dis. 2018 Mar 14;9(3):397. doi: 10.1038/s41419-018-0427-y (PMC5852046; doi:10.1038/s41419-018-0427-y)
Supplement: Supplementary file 2 — Table S1(PDF 360 kb) [file 41419_2018_427_MOESM2_ESM.pdf]

Probe	System	Name	Description	LPS A	LPS B	LPS C	LPS+MYC A	LPS+MYC B	LPS+MYC C	P value	Fold Change
A_51_P112355	NM_018738	Igtp	Interferon gamma induced GTPase	0.1607	0.7470	1.4270	-0.5954	-1.3120	-1.2121	0.00E+00	0.2836
A_55_P2114953	NM_011909	Usp18	Ubiquitin specific peptidase 18	0.8523	0.9878	1.7421	0.5391	-1.3649	-0.9769	0.00E+00	0.2882
A_51_P112355	NM_018738	Igtp	Interferon gamma induced GTPase	0.1037	0.7102	1.4358	-0.4592	-1.3011	-1.2270	3.92E-07	0.2982
A_51_P112355	NM_018738	Igtp	Interferon gamma induced GTPase	0.0837	0.6939	1.4135	-0.5021	-1.2144	-1.3127	0.00E+00	0.2993
A_51_P112355	NM_018738	Igtp	Interferon gamma induced GTPase	0.1375	0.7182	1.4463	-0.3965	-1.2288	-1.2063	3.92E-07	0.3054
A_51_P112355	NM_018738	Igtp	Interferon gamma induced GTPase	0.1501	0.6873	1.4497	-0.4263	-1.2079	-1.1430	7.84E-07	0.3103
A_51_P112355	NM_018738	Igtp	Interferon gamma induced GTPase	0.1309	0.7202	1.3765	-0.4425	-1.1733	-1.2199	7.84E-07	0.3104
A_51_P112355	NM_018738	Igtp	Interferon gamma induced GTPase	0.1050	0.6743	1.3906	-0.4948	-1.2290	-1.1335	7.84E-07	0.3130
A_51_P112355	NM_018738	Igtp	Interferon gamma induced GTPase	0.1398	0.7003	1.3984	-0.3405	-1.2099	-1.2207	7.84E-07	0.3143
A_52_P670026	NM_021384	Rsad2	radical S-adenosyl methionine domain containing 2	0.7112	0.2806	1.3274	0.5668	-2.0188	-1.2336	3.92E-07	0.3146
A_51_P112355	NM_018738	Igtp	Interferon gamma induced GTPase	0.1267	0.7302	1.3077	-0.4275	-1.2082	-1.1764	7.84E-07	0.3167
A_51_P112355	NM_018738	Igtp	Interferon gamma induced GTPase	0.0914	0.7087	1.3816	-0.3752	-1.2360	-1.0664	1.18E-06	0.3254
A_52_P186937	NM_020557	Tyki	Thymidylate kinase family LPS-inducible	0.9568	0.0840	0.9786	0.5701	-1.8496	-1.3197	3.92E-07	0.3440
A_51_P359570	NM_010501	Ifit3	Interferon-induced protein with tetratricopeptide repeats 3	-0.3501	-0.3977	0.1812	-0.6658	-2.1139	-2.2685	3.53E-06	0.3551
A_55_P2056521	NM_019647	Rpl21	ribosomal protein L21	0.2118	-0.2851	-0.7696	-1.7876	-1.6301	-1.5501	7.45E-06	0.3856
A_55_P1999805	A_55_P1999805	Gm8172	ribosomal protein S6 pseudogene	0.0112	-0.0746	-0.4504	-1.5596	-1.6426	-1.2463	1.69E-05	0.4029
A_55_P2004370	NM_009802	Car6	Carbonic anhydrase	-0.2242	-0.3221	-0.1970	-2.0947	-1.3996	-1.1003	2.82E-05	0.4107
A_55_P2082609	NAP113743-1	Unknown	Unknown	0.4717	0.2331	0.4862	-1.3403	-0.9261	-0.3281	3.41E-05	0.4170
A_55_P2072841	A_55_P2072841	Rpl17-ps7	ribosomal protein L17, pseudogene 7	0.2386	-0.6234	-1.0117	-1.4292	-1.9996	-1.7231	1.72E-05	0.4199
A_55_P2145257	NAP102544-1	Unknown	Unknown	0.0070	-0.3510	-0.3521	-1.8141	-1.5589	-1.0757	3.37E-05	0.4202
A_55_P1993758	NM_021552	Nsa2	NSA2 ribosome biogenesis homolog	0.4270	0.2565	0.5862	-1.2988	-0.9176	-0.1992	4.35E-05	0.4268
A_55_P1975447	NAP111403-1	Unknown	Unknown	0.3785	0.2407	0.3713	-1.3056	-1.0796	-0.3057	4.39E-05	0.4272
A_55_P1998461	NM_001002239	Rpl17	Similar to Rpl17 protein	0.1612	-0.6754	-1.0677	-1.4722	-2.0383	-1.7374	2.66E-05	0.4287
A_55_P2066578	BC010546	Ifi204	Interferon inducible gene 204	-1.0596	-0.5439	0.5016	-1.2622	-1.6895	-1.7820	8.62E-05	0.4321
A_55_P1998416	NM_001271676	Ifi47	Interferon inducible gene 47	0.2864	-0.1034	1.0731	-0.0273	-1.3795	-0.9344	6.43E-05	0.4355
A_66_P111689	XM_001003736	Gm9372	ribosomal S28 homologue	0.0845	-0.7786	-1.0878	-1.3637	-2.1231	-1.8681	2.47E-05	0.4380
A_55_P2008567	NM_009096	Rps6	Ribosomal protein S6	-0.0458	-0.1516	-0.4626	-1.4413	-1.6427	-1.1480	3.84E-05	0.4381
A_55_P2145531	NM_138670	Mpst	Mercaptopyruvate sulfurtransferase	-0.1022	0.3645	-0.0793	-1.4314	-1.0264	-0.8576	6.07E-05	0.4456
A_66_P111433	A_66_P111433	Gm6027	Predicted gene	0.1874	0.2663	0.4242	-1.0450	-1.1410	-0.4035	6.43E-05	0.4488
A_55_P2171746	NAP112785-1	Unknown	Unknown	0.3462	0.3673	0.7161	-1.1413	-0.8775	-0.0003	9.25E-05	0.4508
A_51_P232858	NM_025974	Rpl14	Transcribed locus, weakly similar to XP_001058222.1 similar to ribosomal	0.2306	-0.0182	-0.7473	-1.3165	-1.4476	-1.2147	6.86E-05	0.4513
A_55_P2072842	A_55_P2072842	Rpl17-ps7	ribosomal protein L17, pseudogene 7	0.1410	-0.7959	-1.0540	-1.4027	-2.0425	-1.6931	4.39E-05	0.4528
A_55_P2151863	A_55_P2151863	Gm7599	psuedogene	0.2921	0.3300	0.7244	-1.2574	-0.8422	0.0203	1.09E-04	0.4532
A_55_P2076134	A_55_P2076134	Rpl21	ribosomal protein L21	0.2298	-0.5884	-1.0100	-1.7043	-1.7352	-1.3431	1.10E-04	0.4544
A_55_P2118740	A_55_P2118740	Gm15616	psuedogene	0.3086	-0.2640	-0.1810	-1.5487	-1.3103	-0.5876	2.00E-04	0.4654
A_66_P112573	NM_020583	Isg20	Interferon stimulated gene 20	-0.0755	-0.4065	0.0335	-1.0965	-1.2884	-1.3590	9.56E-05	0.4670
A_55_P2130855	ENSMUST00000110798	Unknown	psuedogene	0.2695	0.1447	0.1482	-1.2595	-1.2008	-0.2717	2.01E-04	0.4671
A_55_P2176688	A_55_P2176688	Gm8748	psuedogene	-0.4024	-1.5197	-1.8409	-2.7707	-2.3636	-1.9202	2.15E-04	0.4674
A_55_P2176212	A_55_P2176212	Unknown	Unknown	0.0133	-1.0401	-1.0725	-2.0974	-2.0339	-1.2587	2.32E-04	0.4675

A_55_P2000062	NM_008390	Irf1	Interferon regulatory factor 1	0.4614	0.3654	0.5388	0.1337	-1.0870	-0.9668	4.11E-05	0.4681
A_51_P327751	NM_008331	Ifit1	Interferon-induced protein with tetratricopeptide repeats 1	0.1783	-0.3763	0.7555	0.2200	-1.7962	-1.1448	1.14E-04	0.4688
A_55_P2149906	XM_001479170	Rps23	ribosomal protein S23	-0.3736	-0.7865	-0.9088	-2.3144	-1.6631	-1.3614	2.77E-04	0.4698
A_55_P2049365	XR_168456	Gm3201	predicted gene	-0.0660	-0.3914	-0.7353	-1.8249	-1.6369	-0.9981	2.44E-04	0.4701
A_55_P1968841	A_55_P1968841	Unknown	Unknown	-0.3270	-1.6776	-2.0255	-2.5976	-2.4526	-2.1723	2.18E-04	0.4783
A_51_P490023	NM_009450	Tubb2a	Tubulin beta 2A, Class IIA	0.2820	0.1295	0.6743	-0.7181	-1.0233	-0.3646	1.19E-04	0.4783
A_55_P2069176	NAP014020-001	Unknown	Unknown	0.0291	-1.1729	-1.2349	-2.0830	-2.0206	-1.4597	3.02E-04	0.4791
A_55_P2159094	NM_019782	Lepre1	Leprecan 1	-0.4979	-0.6018	-0.8290	-1.7286	-1.6723	-1.7053	1.32E-04	0.4799
A_55_P2155572	A_55_P2155572	Rpl17-ps7	ribosomal protein L17, pseudogene 7	-0.2061	-1.5401	-1.6993	-2.0558	-2.5140	-2.0492	1.68E-04	0.4803
A_66_P140976	NM_025586	Rpl15	Ribosomal protein L15	0.4778	0.6141	0.1387	-0.7343	-0.7794	-0.4179	1.67E-04	0.4816
A_66_P133993	XM_286185	Rpl11	60S ribosomal protein L11	0.5249	-0.4662	-0.8485	-1.0146	-1.5280	-1.3969	1.21E-04	0.4830
A_55_P2154782	NAP065441-1	Unknown	Unknown	0.3134	-0.1269	-0.6768	-1.1770	-1.3398	-1.1197	1.90E-04	0.4834
A_55_P1998776	A_55_P1998776	Eef1a1	Eukaryotic translation elongation factor1 alpha-like	-0.2983	-0.1824	0.6169	-2.1793	-0.9729	0.1503	1.32E-04	0.4843
A_55_P2015177	A_55_P2015177	Nap1L1	Nucleosome assembly protein 1-like-1	0.6039	0.6183	0.8712	-0.6299	-0.7174	0.3209	2.63E-04	0.4863
A_55_P1978770	NM_001256311	Unknown	Predicted protein	0.0958	-0.0451	0.0492	-1.1880	-1.0145	-0.7982	2.31E-04	0.4885
A_55_P2027906	NM_021552	Nsa2	NSA2 ribosome biogenesis homolog	0.4976	0.4984	0.9130	-0.7255	-0.5782	0.1294	2.64E-04	0.4905
A_55_P2178023	A_55_P2178023	Unknown	Unknown	0.0102	-1.0217	-1.1999	-1.9336	-1.9730	-1.3876	3.94E-04	0.4905
A_55_P2029643	XM_003946058	Nsa2	NSA2 ribosome biogenesis homolog	0.2463	0.3259	0.7209	-0.8954	-0.8864	0.0076	2.54E-04	0.4923
A_55_P2040031	NAP062632-1	Unknown	Unknown	-0.1294	-0.6181	-0.2148	-2.1620	-1.3810	-0.4837	5.32E-04	0.4926
A_66_P107734	DV047974	Rpl17	60S ribosomal protein L17	-0.3100	-1.5789	-1.6528	-2.0877	-2.4850	-2.0319	3.01E-04	0.4928
A_55_P1993761	NM_021552	Nsa2	NSA2 ribosome biogenesis homolog	0.3150	0.2918	0.5862	-0.9315	-0.8962	-0.0339	2.88E-04	0.4937
A_55_P2032559	XR_142050	Gm5075	Predicted gene, EG432725	-0.1131	-1.0053	-1.2380	-2.1444	-1.8128	-1.4360	5.36E-04	0.4958
A_55_P2008691	A_55_P2008691	Gm6392	Pseudogene	-0.3264	-0.2573	0.4726	-1.9909	-1.1204	-0.0189	3.32E-04	0.4978
A_55_P2109599	NAP058895-1	Unknown	Unknown	0.2296	0.3568	0.7202	-0.9485	-0.7907	0.0363	3.28E-04	0.4989
A_55_P1995127	A_55_P1995127	Unknown	Unknown	-0.1417	-0.8646	-1.3919	-2.1055	-1.7365	-1.5607	5.24E-04	0.4995
A_55_P2032167	NM_008327	Ifi202b	Interferon activated gene 202B	0.2316	0.4323	0.8636	-0.0226	-1.0109	-0.4356	1.72E-04	0.5004
A_55_P2044843	NAP102896-1	Unknown	Unknown	-0.6344	-1.2842	-1.2322	-2.2736	-2.1801	-1.6518	5.60E-04	0.5053
A_55_P1961014	NM_009150	Selenbp1	Selenium binding protein 1	0.8737	0.6340	0.7517	-0.6667	-0.1693	0.1415	6.42E-04	0.5053
A_51_P262171	NM_008326	Irgm	Immunity-related GTPase family, M	-0.7501	-0.0025	0.4543	-0.7991	-1.1845	-1.2608	2.81E-04	0.5063
