# Supplementary material for: Inhibition of Sec61-dependent translocation by mycolactone uncouples the integrated stress response from ER stress, driving cytotoxicity via translational activation of ATF4
Source: Cell Death Dis. 2018 Mar 14;9(3):397. doi: 10.1038/s41419-018-0427-y (PMC5852046; doi:10.1038/s41419-018-0427-y)
Supplement: Supplementary file 3 — Table S2(PDF 401 kb) [file 41419_2018_427_MOESM3_ESM.pdf]

| Probe | System | Gene Name | Description | LPS A | LPS B | LPS C | LPS+MYC A | LPS+MYC B | LPS+MYC C | P value | Fold Change |
|---|---|---|---|---|---|---|---|---|---|---|---|
| A_55_P2016459 | AK156907 | Cxcl10 | C-X-C motif chemokine 10 | -1.6316 | -2.7873 | -3.5608 | -0.0912 | 0.0039 | -0.5296 | 0.00E+00 | 5.4803 |
| A_51_P472901 | NM_008577 | Slc3a2 | Solute carrier family 3 (activators of dibasic and neutral amino acid transport), member 2 | -0.0053 | -0.0982 | 0.3439 | 2.6278 | 2.0382 | 2.3511 | 0.00E+00 | 4.7862 |
| A_55_P2115442 | NM_053109 | Clec2d | C-type lectin domain family 2, member d | -2.1319 | -2.5187 | -3.4025 | -0.4584 | -0.7713 | -0.7941 | 0.00E+00 | 4.0272 |
| A_55_P2105858 | NM_030693 | Atf5 | Activating transcription factor 5 | -0.1977 | -1.1628 | -0.3777 | 1.0697 | 0.7450 | 1.3211 | 3.53E-06 | 3.0837 |
| A_51_P519251 | NM_019738 | NupR1 | nuclear protein 1 | 0.2626 | -1.4497 | -1.3744 | 1.9072 | 0.0202 | 0.2095 | 2.74E-06 | 2.9611 |
| A_55_P1960936 | A_55_P1960936 | Accn1 | Accn1/Chr2 var 3 trans-spliced mRNA sequence | -2.5223 | -1.7763 | -0.4156 | -0.8481 | 0.1065 | 0.7030 | 1.45E-05 | 2.9455 |
| A_51_P241861 | NM_016921 | Tcirg1 | T-cell, immune regulator 1, ATPase, H+ transporting, lysosomal V0 protein A3 | -0.5982 | -1.1494 | -0.2915 | 0.9865 | 0.4559 | 1.1728 | 1.37E-05 | 2.9312 |
| A_55_P1954092 | BB713741 | Unknown | RIKEN full-length enriched, 2 cells egg Mus musculus cDNA clone B020049A06 3', mRNA sequence. | -2.5033 | -1.7437 | -0.3230 | -0.7664 | 0.0602 | 0.7295 | 1.80E-05 | 2.8901 |
| A_55_P2044932 | NM_030720 | Gpr84 | G protein-coupled receptor 84 | 0.2745 | 0.7347 | 1.5417 | 2.3656 | 2.2467 | 2.4230 | 4.31E-05 | 2.8183 |
| A_55_P2071952 | NM_178909 | Wdr92 | WD repeat domain 92 | -2.7097 | -1.8913 | -0.1521 | -1.0305 | -0.0763 | 0.7717 | 2.27E-05 | 2.7753 |
| A_55_P2029746 | AK132033 | | non coding RNA | -2.5927 | -1.7490 | -0.0373 | -0.8172 | -0.0147 | 0.8516 | 3.21E-05 | 2.7630 |
| A_51_P323880 | ENSMUST00000082405 | MT-CO2 | mitochondrially encoded cytochrome C oxidase II | -5.2995 | -4.9392 | -4.4504 | -3.6812 | -3.4198 | -3.2211 | 4.31E-05 | 2.7428 |
| A_65_P07196 | ENSMUST00000082409 | MT-CO3 | mitochondrially encoded cytochrome C oxidase III | -5.2556 | -4.5775 | -4.3923 | -3.5365 | -3.4753 | -2.8483 | 4.43E-05 | 2.7418 |
| A_55_P2140212 | TC1616199 | HypF | Hydrogenase maturation factor F, partial | -2.6135 | -1.7556 | -0.1961 | -0.9143 | -0.0374 | 0.7447 | 4.39E-05 | 2.7373 |
| A_55_P1954724 | XR_104972 | Gm20186 | predicted gene | -2.4893 | -1.6594 | -0.2415 | -0.7631 | 0.0612 | 0.6641 | 5.21E-05 | 2.7336 |
| A_51_P464238 | NM_009716 | Atf4 | Activating transcription factor 4 | -1.3220 | -1.5070 | -1.4912 | 0.0339 | -0.0987 | 0.0718 | 2.04E-05 | 2.7177 |
| A_55_P1957213 | AK019465 | YBox3 | Y Box 3 protein, DNA binding | -2.4754 | -1.6591 | -0.2449 | -0.7790 | 0.0294 | 0.6610 | 6.74E-05 | 2.6950 |
| A_51_P183812 | NM_011410 | Slfn4 | Schlafen 4 | -3.0403 | -3.0561 | -3.7383 | -1.6475 | -1.9020 | -2.0595 | 2.12E-05 | 2.6547 |
| A_55_P1984556 | NM_011331 | Ccl12 | Chemokine (C-C motif) ligand 12 | -0.7961 | -1.1332 | -2.4540 | 0.0578 | -0.0115 | -0.2458 | 3.21E-05 | 2.6291 |
| A_51_P144770 | BC016099 | Unnown | hypothetical protein | -1.4753 | -1.3236 | -0.9332 | 0.0652 | 0.0281 | 0.3419 | 7.76E-05 | 2.6191 |
| A_55_P2036240 | AK141540 | | non coding RNA | -2.6389 | -2.1206 | -0.7585 | -1.4241 | -0.3656 | 0.3490 | 8.11E-05 | 2.5653 |
| A_51_P315595 | ENSMUST00000082421 | mt-Cytb | mitochondrially encoded cytochrome b | -3.7987 | -3.1146 | -3.2954 | -1.9391 | -1.8432 | -2.3944 | 8.46E-05 | 2.5386 |
| A_55_P1979728 | NM_009716 | Atf4 | Activating transcription factor 4 | -1.0190 | -0.8279 | 0.3252 | 0.5817 | 0.6060 | 1.3092 | 1.40E-04 | 2.5307 |
| A_66_P101646 | ENSMUST00000082402 | mt-Co1 | mitochondrially encoded cytochrome c oxidase I | -3.1877 | -2.4049 | -2.2326 | -1.3252 | -1.3063 | -1.2408 | 1.90E-04 | 2.4926 |
| A_55_P2088720 | AK035396 | | Non coding RNA | -2.7010 | -1.9669 | -0.4822 | -1.4985 | -0.1855 | 0.4400 | 1.03E-04 | 2.4657 |
| A_55_P1955279 | NM_001029936 | Specc1 | sperm antigen with calponin homology and coiled-coil domains 1 | -2.3629 | -2.6486 | -2.4667 | -1.7564 | -1.3788 | -0.4371 | 1.27E-04 | 2.4657 |
| A_51_P409429 | NM_146217 | Aars | Alanyl-tRNA synthetase | 1.2115 | -0.3746 | 1.5659 | 2.1090 | 1.4084 | 2.7714 | 9.29E-05 | 2.4543 |
| A_51_P187901 | NM_024193 | Nol5a | Nucleolar protein 5A | -1.2210 | -1.7217 | -1.3614 | 0.0891 | -0.5120 | -0.0206 | 1.26E-04 | 2.4400 |
| A_55_P1985519 | ENSMUST00000036360 | | Uncharacterized protein C1orf54 homolog | -3.6013 | -3.8256 | -3.6363 | -2.6256 | -2.5064 | -2.0828 | 1.23E-04 | 2.4332 |
| A_52_P321318 | ENSMUST00000082408 | mt-Atp6 | mitochondrially encoded ATP synthase 6 | -5.3468 | -4.7780 | -4.6154 | -3.7272 | -3.4776 | -3.6924 | 1.94E-04 | 2.4301 |
| A_52_P200617 | NM_172015 | Iars | Isoleucine-tRNA synthetase | -0.2414 | -0.5074 | 0.7709 | 0.9852 | 0.8557 | 2.0146 | 1.98E-04 | 2.4247 |
| A_55_P1959064 | NM_001083925 | Oas1b | 2'-5' oligoadenylate synthetase 1B | -1.8631 | -2.3084 | -2.1525 | -0.5707 | -1.0114 | -0.9094 | 1.09E-04 | 2.4242 |
| A_55_P1968606 | NM_194341 | Ap1gbp1 | AP1 gamma subunit binding protein 1 | -3.3559 | -3.5803 | -3.4090 | -2.3418 | -2.2886 | -1.9102 | 1.50E-04 | 2.4086 |
| A_55_P2053398 | ENSMUST00000078451 | Lirb3 | Cell-surface glycoprotein p91 | -3.2986 | -3.4302 | -3.2229 | -2.2302 | -2.1930 | -1.7738 | 2.04E-04 | 2.3810 |
| A_66_P115406 | NM_008774 | Pabpc1 | poly(A) binding protein, cytoplasmic 1 | -3.3647 | -2.3729 | -0.3448 | -1.5022 | -1.1307 | 0.2943 | 1.14E-04 | 2.3750 |
| A_55_P1965827 | NM_011664 | Ubb | ubiquitin B | 0.4276 | 0.4837 | 0.7989 | 2.1964 | 1.3800 | 1.8722 | 2.95E-04 | 2.3720 |
| A_55_P1956063 | NM_026701 | Pbld | Phenazine biosynthesis-like protein domain containing | -3.6357 | -3.7655 | -3.4152 | -2.6100 | -2.4831 | -2.0251 | 2.50E-04 | 2.3501 |
| A_55_P2065731 | NM_027519 | Medag | mesenteric estrogen dependent adipogenesis | -3.3235 | -3.5193 | -3.3535 | -2.2805 | -2.3299 | -1.8907 | 2.43E-04 | 2.3484 |
| A_55_P2060444 | NM_026511 | Apopt1 | apoptogenic, mitochondrial 1 | -3.5614 | -3.8376 | -3.7146 | -2.7091 | -2.5557 | -2.1771 | 2.05E-04 | 2.3358 |
| A_55_P2114269 | NM_001100116 | Fam177a1 | Protein FAM177A1 | -3.3181 | -3.5408 | -3.3898 | -2.3818 | -2.2969 | -1.9091 | 2.36E-04 | 2.3300 |
| A_55_P2137941 | NM_052823 | Fxyd2 | FXYD domain-containing ion transport regulator 2 | -1.5222 | -0.9954 | -0.5964 | -0.0101 | 0.2593 | 0.2813 | 4.24E-04 | 2.3211 |

| | | | | | | | | | | | |
|---|---|---|---|---|---|---|---|---|---|---|---|
| A_55_P1972263 | BC002231 | Nop56 | nucleolar protein 5A | -1.8717 | -1.7589 | -1.0111 | -0.4048 | -0.8110 | 0.2091 | 4.78E-04 | 2.3160 |
| A_55_P2117487 | A_55_P2117487 | PabpC1 | Predicted poly(A) binding protein, cytoplasmic 1 (Pabpc1), transcript variant X1 | -3.4481 | -2.5041 | -0.4726 | -1.6621 | -1.3040 | 0.1735 | 1.58E-04 | 2.3145 |
| A_55_P2028600 | NM_022410 | Myh9 | Myosin, heavy polypeptide 9, non-muscle | 0.2964 | 0.3734 | 1.8326 | 1.3652 | 1.6218 | 3.1155 | 2.52E-04 | 2.2974 |
| A_55_P2143075 | NM_019639 | Ubc | Ubiquitin C | -1.4384 | -0.4440 | 0.5495 | 0.2421 | 0.3567 | 1.6597 | 5.38E-04 | 2.2928 |
| A_55_P2069935 | NM_052823 | Fxyd2 | FXYD domain-containing ion transport regulator 2 | -1.6334 | -0.8369 | -0.3250 | 0.0641 | 0.3521 | 0.3769 | 5.93E-04 | 2.2912 |
| A_55_P2034928 | NM_001037925 | LOC625360 | Similar to 2-cell-stage, variable group, member 3 | -1.0151 | -0.6031 | -1.2157 | 0.0806 | 0.4873 | 0.1826 | 2.63E-04 | 2.2891 |
| A_55_P2092085 | NM_001190327 | Tmsb1 | thymosin, beta 10 | -2.3543 | -2.4158 | -2.5368 | -1.3843 | -1.1134 | -1.2811 | 2.79E-04 | 2.2596 |
| A_55_P2027819 | NM_018860 | Rpl41 | Ribosomal protein L41 | -3.0307 | -2.5386 | -1.7658 | -1.3415 | -1.3593 | -1.1209 | 8.06E-04 | 2.2519 |
| A_51_P228768 | NM_011409 | Slfn3 | schlafen 3 | -0.5622 | -1.5699 | -2.6861 | 0.0607 | -0.6029 | -0.7722 | 1.14E-04 | 2.2469 |
| A_55_P2153067 | NM_029816 | Orf67 | Uncharacterized protein | -3.3696 | -3.5388 | -3.1006 | -2.3864 | -2.3443 | -1.7905 | 5.12E-04 | 2.2387 |
| A_55_P1973347 | AK149472 | | Mus musculus adult male liver tumor cDNA, RIKEN full-length enriched library, clone:C730020H24 product:hypothetical protein, | -2.0797 | -1.5257 | -0.6901 | -0.7295 | -0.1857 | 0.1013 | 6.87E-04 | 2.2354 |
| A_66_P100586 | ENSMUST00000101511 | | Antisense RNA | -1.0364 | -0.9016 | -1.8208 | -0.0796 | 0.0032 | -0.2083 | 2.43E-04 | 2.2315 |
| A_52_P533304 | NM_173363 | Eif5 | Eukaryotic translation initiation factor 5 | -0.4459 | 0.5538 | 0.6581 | 0.6871 | 1.5645 | 1.9861 | 5.21E-04 | 2.2304 |
| A_52_P684378 | NM_008160 | Gpx1 | glutathione peroxidase 1 | -1.9068 | -2.0263 | -1.5185 | -0.3076 | -0.8689 | -0.8071 | 5.94E-04 | 2.2284 |
| A_52_P533146 | NM_007837 | Ddit3 | DNA-damage inducible transcript 3 | -0.6033 | -1.8750 | -1.8215 | 0.3889 | -0.7750 | -0.4476 | 1.62E-04 | 2.2275 |
| A_51_P458384 | NM_175121 | Slc38a2 | solute carrier family 38, member 2 | -0.3734 | -0.2465 | 0.2112 | 0.5738 | 0.9514 | 1.5271 | 5.86E-04 | 2.2248 |
| A_55_P1969874 | A_55_P1969874 | | unknown | -3.0765 | -3.3372 | -3.1908 | -2.1692 | -2.1889 | -1.8093 | 5.06E-04 | 2.2125 |
| A_55_P2034740 | NM_144886 | Exosc2 | exosome component 2 | -3.1149 | -3.3012 | -2.8527 | -2.0242 | -2.1444 | -1.6678 | 6.37E-04 | 2.2101 |
| A_52_P49601 | NM_010239 | Fth1 | ferritin heavy chain 1 | -3.6073 | -2.6075 | -1.7070 | -2.0875 | -1.5291 | -0.8733 | 7.86E-04 | 2.2098 |
| A_55_P2072373 | NM_010833 | Msn | Moesin | 1.1105 | 0.1418 | 1.6148 | 1.8173 | 1.6029 | 2.8785 | 4.45E-04 | 2.2097 |
| A_55_P1998811 | A_55_P1998811 | Gm3430 | predicted gene 3430 | -2.6999 | -2.2589 | -0.7320 | -2.0509 | -0.4783 | 0.2672 | 2.86E-04 | 2.2083 |
| A_55_P2105843 | A_55_P2105843 | Gm2371 | predicted gene 2371 | -2.5053 | -2.0118 | -0.7729 | -1.7181 | -0.3863 | 0.2370 | 3.97E-04 | 2.2052 |
| A_55_P2090505 | A_55_P2090505 | Gm4382 | predicted gene 4382 | -2.6708 | -2.1332 | -0.5827 | -1.6043 | -0.5273 | 0.1676 | 4.54E-04 | 2.2051 |
| A_52_P305851 | NM_025880 | LOC668661 | uncharacterized protein LOC668661 | -1.3692 | -1.4960 | -1.3929 | -0.3675 | -0.1727 | -0.2972 | 4.33E-04 | 2.2042 |
| A_55_P2078994 | AK136409 | Lrrc31 | leucine rich repeat containing 31 | -3.5165 | -3.7478 | -3.6442 | -2.7632 | -2.5610 | -2.1735 | 5.02E-04 | 2.1992 |
| A_51_P241457 | NM_013532 | Lilrb4 | leukocyte immunoglobulin-like receptor, subfamily B, member 4 | -0.9471 | -0.6380 | -0.6690 | 0.6618 | 0.1984 | 0.2741 | 7.59E-04 | 2.1878 |
| A_55_P1999902 | ENSMUST00000107229 | Pip5k1a | phosphatidylinositol-4-phosphate 5-kinase, type 1 alpha | -1.4269 | -2.7517 | -1.7170 | -0.1085 | -1.3690 | -1.0323 | 4.15E-04 | 2.1864 |
| A_55_P2151601 | NM_010156 | Samd9l | sterile alpha motif domain containing 9-like | -1.6058 | -1.9609 | -1.9021 | -0.6841 | -0.8969 | -0.5405 | 6.01E-04 | 2.1671 |
| A_55_P2081432 | A_55_P2081432 | Gm8953 | 2-cell-stage, variable group, member 3-like pseudogene | -0.7985 | -0.9009 | -1.2749 | -0.0376 | 0.1742 | 0.2316 | 4.40E-04 | 2.1647 |
| A_55_P2158281 | A_55_P2158281 | | Unknown | -2.9493 | -3.2837 | -3.2130 | -2.1236 | -2.2001 | -1.7880 | 6.60E-04 | 2.1606 |
| A_55_P2105512 | NM_024193 | Nol5a | Nucleolar protein 5A | -1.9749 | -1.9870 | -1.3134 | -0.8010 | -1.0791 | -0.0731 | 1.03E-03 | 2.1545 |
| A_55_P2165234 | NM_001081961 | 2300005B03Rik | Secreted Ly6/uPAR related protein 2 | -2.7877 | -0.3561 | -0.5867 | -0.2824 | 0.1899 | -0.3204 | 2.16E-03 | 2.1523 |
| A_51_P246317 | NM_008630 | Mt2 | Mouse metallothionein 2 | -1.4964 | -2.2697 | -0.7406 | 0.4140 | -0.9349 | -0.6774 | 8.65E-04 | 2.1477 |
| A_55_P1953475 | A_55_P1953475 | | Unknown | 0.6882 | 0.6383 | 0.8649 | 2.2078 | 1.4878 | 1.8022 | 9.09E-04 | 2.1467 |
| A_55_P2159740 | NM_001033488 | Mfsd2b | major facilitator superfamily domain containing 2B | -3.3236 | -3.6094 | -3.5707 | -2.6966 | -2.4291 | -2.0724 | 6.02E-04 | 2.1463 |
| A_55_P2093940 | NM_001033451 | Zfp408 | Zinc finger protein 408 | -3.1596 | -3.4810 | -3.3841 | -2.4077 | -2.3652 | -1.9475 | 7.14E-04 | 2.1457 |
| A_51_P385099 | NM_013693 | Tnf | Tumor necrosis factor | -0.8158 | -0.6224 | 0.0185 | 0.5834 | 0.5959 | 0.6717 | 1.20E-03 | 2.1291 |
| A_65_P05358 | ENSMUST00000082392 | mt-Nd1 | mitochondrially encoded NADH dehydrogenase 1 | -4.5769 | -4.4143 | -3.8298 | -3.7003 | -2.9577 | -2.8987 | 9.98E-04 | 2.1260 |
| A_55_P1957459 | NM_013532 | Lilrb4 | leukocyte immunoglobulin-like receptor, subfamily B, member 4 | -1.1091 | -0.5213 | -0.1747 | 0.8095 | 0.1989 | 0.4400 | 1.68E-03 | 2.1206 |
| A_55_P2136880 | NM_008654 | Ppp1r15a | protein phosphatase 1, regulatory (inhibitor) subunit 15A | -0.6415 | -1.0140 | -0.7233 | 0.5619 | 0.0724 | 0.2376 | 8.10E-04 | 2.1193 |

| | | | | | | | | | | | | |
|---|---|---|---|---|---|---|---|---|---|---|---|---|
| A_55_P2063736 | NM_008147 | Gp49a | glycoprotein 49 A | -1.0460 | -0.7392 | -0.8826 | 0.5623 | -0.0060 | 0.0213 | 1.07E-03 | 2.1167 |
| A_55_P1989816 | NM_009565 | Zbtb7b | Zinc finger and BTB domain containing 7B | -1.1653 | -0.9173 | -0.4408 | 0.0967 | 0.1191 | 0.4995 | 1.31E-03 | 2.1134 |
| A_51_P385099 | NM_013693 | Tnf | Tumor necrosis factor | -0.8466 | -0.6107 | -0.0127 | 0.5608 | 0.5408 | 0.6590 | 1.37E-03 | 2.1094 |
| A_52_P683336 | NM_145229 | AY074887 | CDNA sequence AY074887 | -0.6999 | -1.5122 | -1.3745 | 0.0788 | -0.1715 | -0.2705 | 4.77E-04 | 2.1060 |
| A_51_P215887 | NM_019698 | Aldh18a1 | Aldehyde dehydrogenase 18 family, member A1 | 0.4278 | -0.1464 | 0.3445 | 1.1880 | 1.1178 | 1.5312 | 9.16E-04 | 2.0999 |
| A_51_P385099 | NM_013693 | Tnf | Tumor necrosis factor | -0.8738 | -0.5853 | -0.0152 | 0.5733 | 0.5448 | 0.6125 | 1.53E-03 | 2.0969 |
| A_55_P1983959 | NM_010240 | Ftl1 | ferritin light chain 1 | -3.6904 | -3.1013 | -2.2105 | -2.5398 | -2.2607 | -1.0058 | 1.27E-03 | 2.0926 |
| A_55_P2006467 | NM_011354 | Serf2 | small EDRK-rich factor 2 | -2.3237 | -1.5916 | -1.3626 | -1.1456 | -0.4208 | -0.5169 | 1.21E-03 | 2.0920 |
| A_51_P385099 | NM_013693 | Tnf | Tumor necrosis factor | -0.8370 | -0.6604 | -0.0543 | 0.5235 | 0.5164 | 0.5999 | 1.48E-03 | 2.0905 |
| A_51_P217463 | NM_009140 | Cxcl2 | chemokine (C-X-C motif) ligand 2 | -0.3217 | -1.6519 | -1.6100 | 0.7337 | -0.4715 | -0.6595 | 2.57E-04 | 2.0880 |
| A_51_P172853 | NM_009841 | Cd14 | CD14 antigen | -0.0604 | 0.4817 | 0.6985 | 1.7812 | 1.2625 | 1.2612 | 1.82E-03 | 2.0874 |
| A_55_P1954925 | NM_177394 | Endov | endonuclease V | -2.8915 | -2.9283 | -3.0182 | -1.9643 | -1.9207 | -1.7680 | 9.97E-04 | 2.0873 |
| A_55_P2108165 | NM_001164289 | Phf11c | PHD finger protein 11C | -1.0908 | -0.9331 | -1.1422 | -0.1238 | -0.0053 | 0.1451 | 1.01E-03 | 2.0860 |
| A_55_P2098450 | NM_177214 | Ascc3l1 | Activating signal cointegrator 1 complex subunit 3-like 1 | -0.0677 | -0.3939 | 0.8375 | 0.5265 | 0.7748 | 2.2537 | 1.03E-03 | 2.0844 |
| A_51_P385099 | NM_013693 | Tnf | Tumor necrosis factor | -0.8846 | -0.5656 | -0.0765 | 0.5382 | 0.5479 | 0.5613 | 1.62E-03 | 2.0821 |
| A_51_P385099 | NM_013693 | Tnf | Tumor necrosis factor | -0.8716 | -0.6782 | -0.1185 | 0.5166 | 0.4429 | 0.5449 | 1.63E-03 | 2.0814 |
| A_51_P385099 | NM_013693 | Tnf | Tumor necrosis factor | -0.8467 | -0.5726 | 0.0145 | 0.5742 | 0.5408 | 0.6519 | 1.69E-03 | 2.0810 |
| A_55_P2109857 | NM_009061 | Rgs2 | Regulator of G-protein signaling 2 | -0.0097 | -0.7692 | -0.6012 | 1.4619 | 0.1048 | 0.2231 | 6.81E-04 | 2.0801 |
| A_51_P328442 | NM_033327 | Zfp423 | Zinc finger protein 423 | -4.2768 | 0.6249 | 1.1446 | 0.0656 | 0.2460 | 0.3417 | 1.97E-03 | 2.0756 |
| A_55_P2185712 | NM_009938 | Copa | Coatomer protein complex subunit alpha | 0.3844 | -0.5498 | 1.0308 | 1.1831 | 0.9052 | 1.9367 | 1.02E-03 | 2.0752 |
| A_55_P2008884 | NM_001003908 | Cltc | Clathrin, heavy polypeptide (Hc) | 0.0676 | -0.6053 | 1.2698 | 0.4562 | 0.7084 | 2.7255 | 4.43E-04 | 2.0744 |
| A_55_P2144126 | A_55_P2144126 | Gm3788 | predicted gene 3788 | -2.5367 | -2.6856 | -2.8255 | -1.9200 | -1.4260 | -1.5472 | 7.88E-04 | 2.0728 |
| A_51_P385099 | NM_013693 | Tnf | Tumor necrosis factor | -0.8698 | -0.5852 | -0.0114 | 0.5423 | 0.5536 | 0.5791 | 1.79E-03 | 2.0664 |
| A_55_P2039110 | ENSMUST00000082396 | mt-Nd2 | mitochondrially encoded NADH dehydrogenase 2 | -4.7745 | -4.3584 | -3.2589 | -3.1977 | -2.8140 | -3.2426 | 2.00E-03 | 2.0646 |
| A_55_P2156394 | NM_001081038 | Btbd16 | BTB (POZ) domain containing 16 | -1.1426 | -1.4648 | -1.2378 | -0.1679 | -0.2919 | -0.2480 | 1.06E-03 | 2.0645 |
| A_51_P265869 | NM_010481 | Hspa9 | Heat shock protein 9 | 0.7469 | 0.5634 | 1.5149 | 2.1706 | 1.4025 | 2.3848 | 1.53E-03 | 2.0623 |
| A_55_P2060111 | ENSMUST00000082407 | mt-Atp8 | mitochondrially encoded ATP synthase 8 | -5.3327 | -4.7749 | -4.5777 | -3.9788 | -3.6067 | -3.9706 | 1.53E-03 | 2.0606 |
| A_55_P1978866 | A_55_P1978866 | LOC100039402, | similar to pol protein, 100039402 | -2.4950 | -1.8973 | -0.7472 | -1.6060 | -0.4773 | 0.0692 | 1.01E-03 | 2.0588 |
| A_55_P2094901 | ENSMUST00000085290 | Phyhd1 | phytanoyl-CoA dioxygenase domain containing 1 | -3.2774 | -3.5169 | -3.3866 | -2.5954 | -2.3954 | -2.0667 | 1.18E-03 | 2.0578 |
| A_55_P2099358 | NM_013742 | Cars | Cysteinyl-tRNA synthetase | 0.6173 | -0.6406 | 0.8150 | 1.6818 | 0.5825 | 1.6470 | 8.97E-04 | 2.0560 |
| A_55_P2403453 | NR_028266 | | non-coding RNA | -3.3365 | -0.0177 | 0.4080 | -0.0559 | 0.1906 | 0.0383 | 6.25E-03 | 2.0558 |
| A_52_P431615 | XM_001000891 | Gm1966 | predicted gene | -1.1346 | -1.2235 | -1.3409 | 0.0220 | -0.3338 | -0.2747 | 1.10E-03 | 2.0527 |
| A_51_P123625 | NM_008392 | Irg1 | immunoresponsive gene 1 | 0.0401 | 0.1296 | 1.2480 | 1.5281 | 1.1875 | 1.8106 | 2.16E-03 | 2.0508 |
| A_55_P2120577 | NM_010828 | Cited2 | Cbp/p300-interacting transactivator, with Glu/Asp-rich carboxy-terminal domain, 2 | 0.3517 | 0.4027 | 0.6936 | 1.7532 | 1.5065 | 1.2949 | 1.51E-03 | 2.0499 |
| A_51_P253803 | NM_001081117 | Mki67 | antigen identified by monoclonal antibody Ki 67 | -1.3086 | -0.5318 | -0.0864 | -0.5275 | 0.3547 | 1.3497 | 1.29E-03 | 2.0485 |
| A_55_P2109486 | NM_001033435 | Milr1 | mast cell immunoglobulin like receptor 1 | -2.8240 | -3.0957 | -2.8023 | -1.9261 | -2.0758 | -1.6192 | 1.45E-03 | 2.0471 |
| A_55_P2081530 | NM_080559 | Sh3bgrl3 | SH3 domain binding glutamic acid-rich protein-like 3 | -0.7186 | -0.7063 | 0.2106 | 0.8269 | 0.4011 | 0.6558 | 2.17E-03 | 2.0459 |
| A_55_P2041265 | NM_144826 | Utp6 | UTP6, small subunit (SSU) processome component, homolog (yeast) | -2.9564 | -3.3426 | -3.3843 | -2.3827 | -2.2931 | -1.9147 | 1.10E-03 | 2.0433 |
| A_51_P123625 | NM_008392 | Irg1 | immunoresponsive gene 1 | 0.0164 | 0.1497 | 1.2853 | 1.5346 | 1.1895 | 1.8129 | 2.38E-03 | 2.0400 |
| A_55_P1956973 | NM_011602 | Tln1 | Talin 1 | -0.5545 | -0.4440 | 0.6086 | 0.2732 | 0.6322 | 1.7893 | 1.57E-03 | 2.0395 |
| A_55_P1973279 | A_55_P1973279 | | Unknown | -3.1812 | -2.5634 | -1.5617 | -1.9823 | -1.6316 | -0.6104 | 1.65E-03 | 2.0382 |
| A_51_P488739 | NM_030701 | Gpr109a | G protein-coupled receptor 109A | -0.4075 | -0.7789 | -1.1796 | 0.8978 | 0.0857 | -0.2677 | 6.43E-04 | 2.0381 |
| A_52_P56751 | NM_008879 | Lcp1 | lymphocyte cytosolic protein 1 | 0.8176 | 0.4437 | 1.8606 | 2.0966 | 1.5471 | 2.5519 | 1.56E-03 | 2.0344 |
| A_51_P123625 | NM_008392 | Irg1 | immunoresponsive gene 1 | 0.0494 | 0.1131 | 1.3048 | 1.4885 | 1.1797 | 1.8723 | 2.24E-03 | 2.0341 |

| | | | | | | | | | | | |
|---|---|---|---|---|---|---|---|---|---|---|---|
| A_51_P123625 | NM_008392 | Irg1 | immunoresponsive gene 1 | 0.0696 | 0.0879 | 1.3208 | 1.4770 | 1.2130 | 1.8515 | 2.29E-03 | 2.0295 |
| A_55_P1974178 | NM_175026 | Pyhin1 | Pyrin and HIN domain family, member 1 | -0.7262 | -1.0448 | -1.8200 | -0.3156 | -0.0228 | -0.1956 | 6.90E-04 | 2.0265 |
| A_51_P385099 | NM_013693 | Tnf | Tumor necrosis factor | -0.8232 | -0.5928 | -0.0405 | 0.5703 | 0.5201 | 0.5053 | 2.25E-03 | 2.0243 |
| A_51_P315555 | NM_027350 | Nars | Asparaginyl-tRNA synthetase | 0.8293 | 0.3524 | 0.9691 | 1.7708 | 1.4447 | 1.9870 | 1.60E-03 | 2.0240 |
| A_51_P385099 | NM_013693 | Tnf | Tumor necrosis factor | -0.8303 | -0.6645 | -0.0527 | 0.5128 | 0.4867 | 0.5037 | 2.18E-03 | 2.0236 |
| A_55_P2061737 | NM_021278 | Tmsb4x | thymosin, beta 4, X chromosome | -1.2584 | -2.4899 | -2.5688 | 0.2231 | -1.9424 | -1.5506 | 4.17E-04 | 2.0219 |
| A_51_P345663 | NM_024281 | Rrbp1 | Ribosome binding protein 1 | -0.1444 | -0.5632 | 0.7428 | 0.6046 | 0.6926 | 1.7834 | 1.63E-03 | 2.0211 |
| A_55_P1977917 | NM_001146311 | Cln3 | ceroid lipofuscinosis, neuronal 3, juvenile (Batten, Spielmeyer-Vogt disease) | -1.1536 | -0.6531 | -0.1001 | 0.7083 | 0.1214 | 0.3061 | 3.03E-03 | 2.0198 |
| A_52_P679860 | NM_025992 | Herc6 | hect domain and RLD 6 | -0.6116 | -1.1002 | -0.5932 | 0.4448 | -0.2849 | 0.5718 | 1.68E-03 | 2.0170 |
| A_55_P2011862 | NM_008653 | Mybpc3 | Myosin binding protein C, cardiac | -2.0433 | -2.3499 | -2.2942 | -1.0962 | -1.3946 | -1.1603 | 1.39E-03 | 2.0168 |
| A_51_P185660 | NM_011338 | Ccl9 | Chemokine (C-C motif) ligand 9 | -0.0887 | -0.3040 | -0.3855 | 1.5511 | 0.3827 | 0.3202 | 1.22E-03 | 2.0149 |
| A_55_P2008879 | NM_001003908 | Cltc | Clathrin, heavy polypeptide (Hc) | 0.1590 | -0.4159 | 0.6799 | 0.5509 | 0.7331 | 2.1706 | 1.53E-03 | 2.0146 |
| A_55_P2113256 | NM_181402 | Parp11 | Poly (ADP-ribose) polymerase family, member 11 | -0.3128 | -1.1534 | -1.6408 | 0.0383 | 0.0643 | -0.1882 | 6.57E-04 | 2.0099 |
| A_51_P123625 | NM_008392 | Irg1 | immunoresponsive gene 1 | 0.0634 | 0.1183 | 1.3241 | 1.5072 | 1.1827 | 1.8327 | 2.61E-03 | 2.0077 |
| A_51_P327295 | NM_021473 | Akr1a1 | aldo-keto reductase family 1, member A1 | -0.3073 | 0.2381 | 0.1777 | 1.0857 | 0.9324 | 1.1040 | 2.23E-03 | 2.0063 |
| A_55_P2082733 | NM_007807 | Cybb | Cytochrome b-245, beta polypeptide | -0.1315 | -0.2319 | 0.5688 | 0.8230 | 0.9934 | 1.4022 | 2.10E-03 | 2.0061 |
| A_55_P1985422 | TC1700876 | Unknown | Erythroid differentiation regulator, partial (43%) | -2.0395 | -1.8954 | -1.4686 | -1.0428 | -0.6531 | -0.6952 | 1.88E-03 | 2.0057 |
| A_55_P2152926 | NM_016776 | Mybbp1a | MYB binding protein (P160) 1a | 0.4729 | -0.5388 | 1.6811 | 1.5497 | 0.8999 | 2.1755 | 9.13E-04 | 2.0046 |
| A_51_P362054 | NM_025983 | Atp5e | ATP synthase, H+ transporting, mitochondrial F1 complex, epsilon subunit | -2.8657 | -1.7359 | -1.7341 | -1.7654 | -0.9112 | -0.6569 | 1.88E-03 | 2.0010 |
