# Supplementary figures and images for: Inhibition of Sec61-dependent translocation by mycolactone uncouples the integrated stress response from ER stress, driving cytotoxicity via translational activation of ATF4
Source: Cell Death Dis. 2018 Mar 14;9(3):397. doi: 10.1038/s41419-018-0427-y (PMC5852046; doi:10.1038/s41419-018-0427-y)

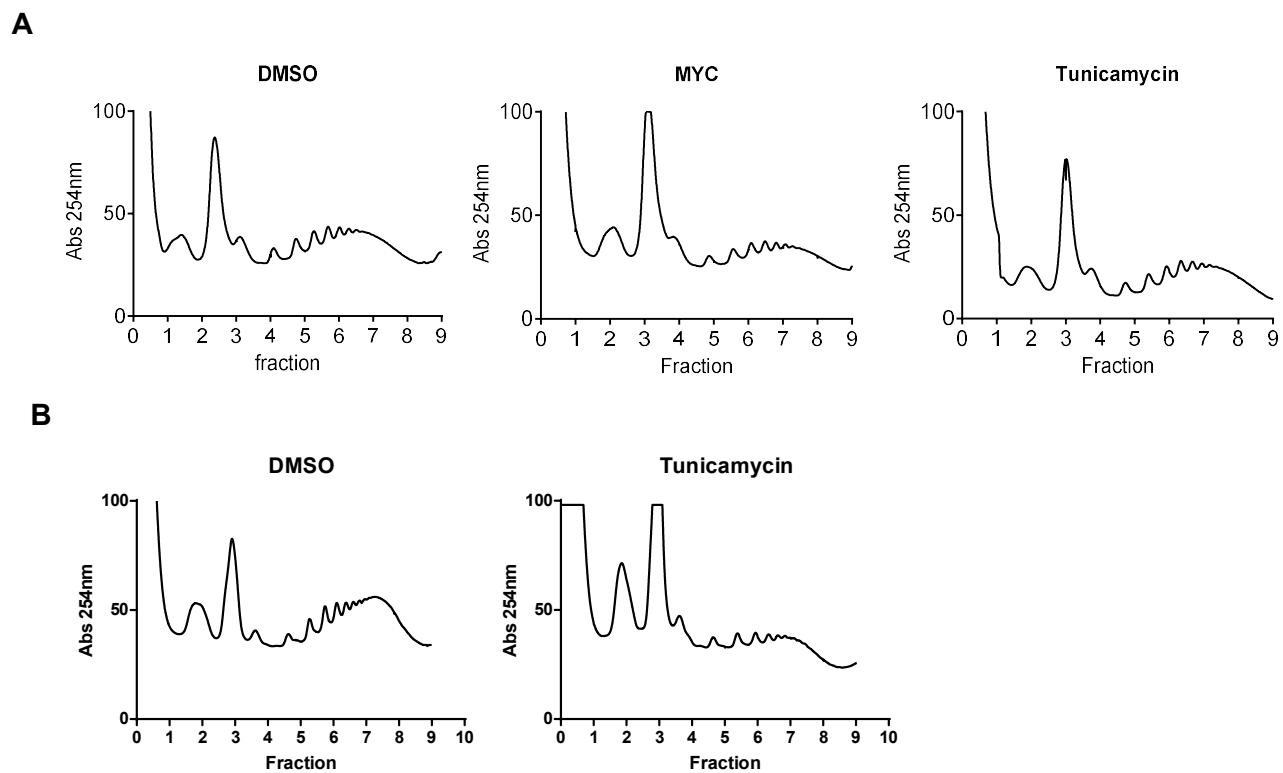

Fig. S1

Supplement: Supplementary file 4 — Figure S1(PDF 220 kb) [file 41419_2018_427_MOESM4_ESM.pdf]

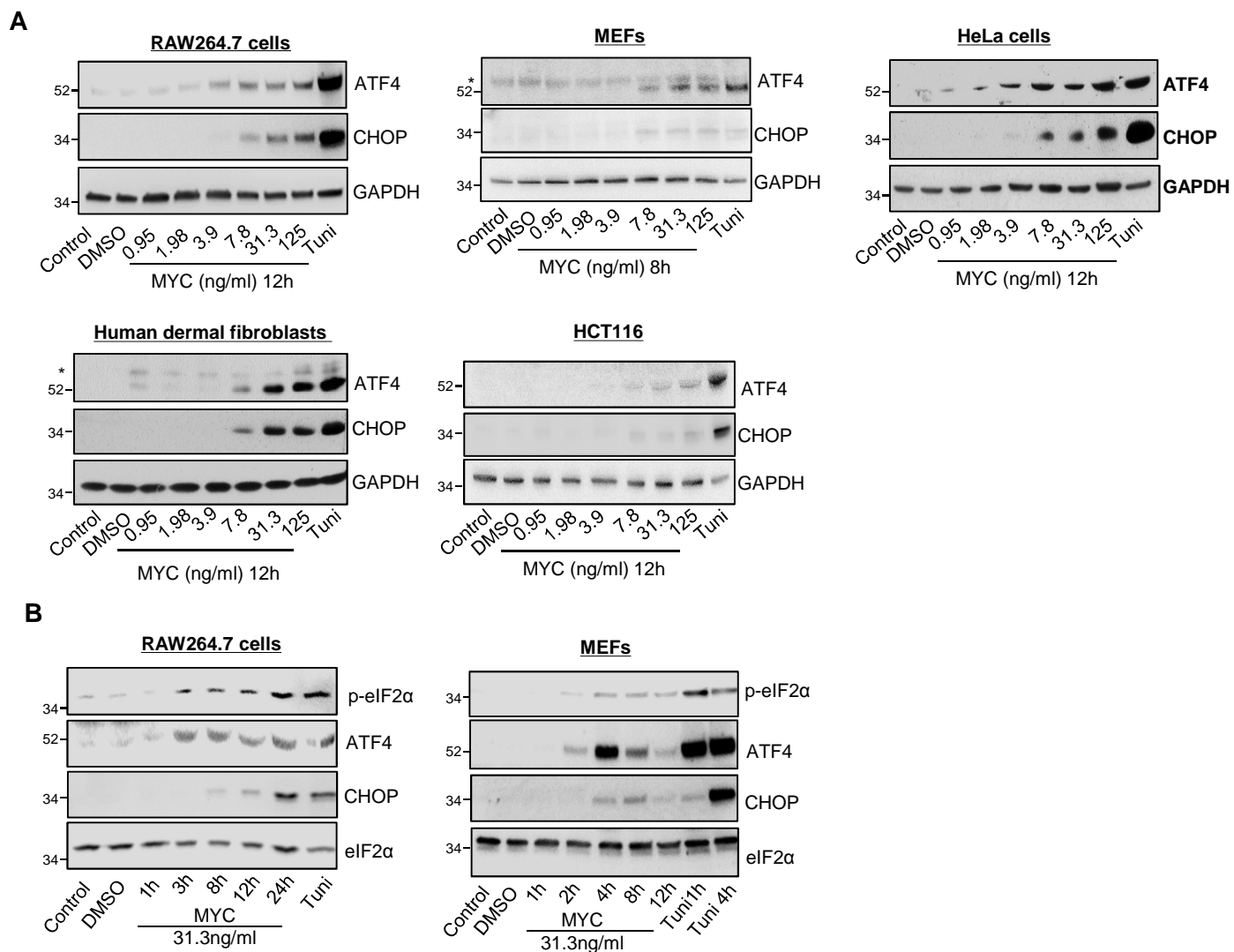

Fig. S2

Supplement: Supplementary file 5 — Figure S2(PDF 714 kb) [file 41419_2018_427_MOESM5_ESM.pdf]

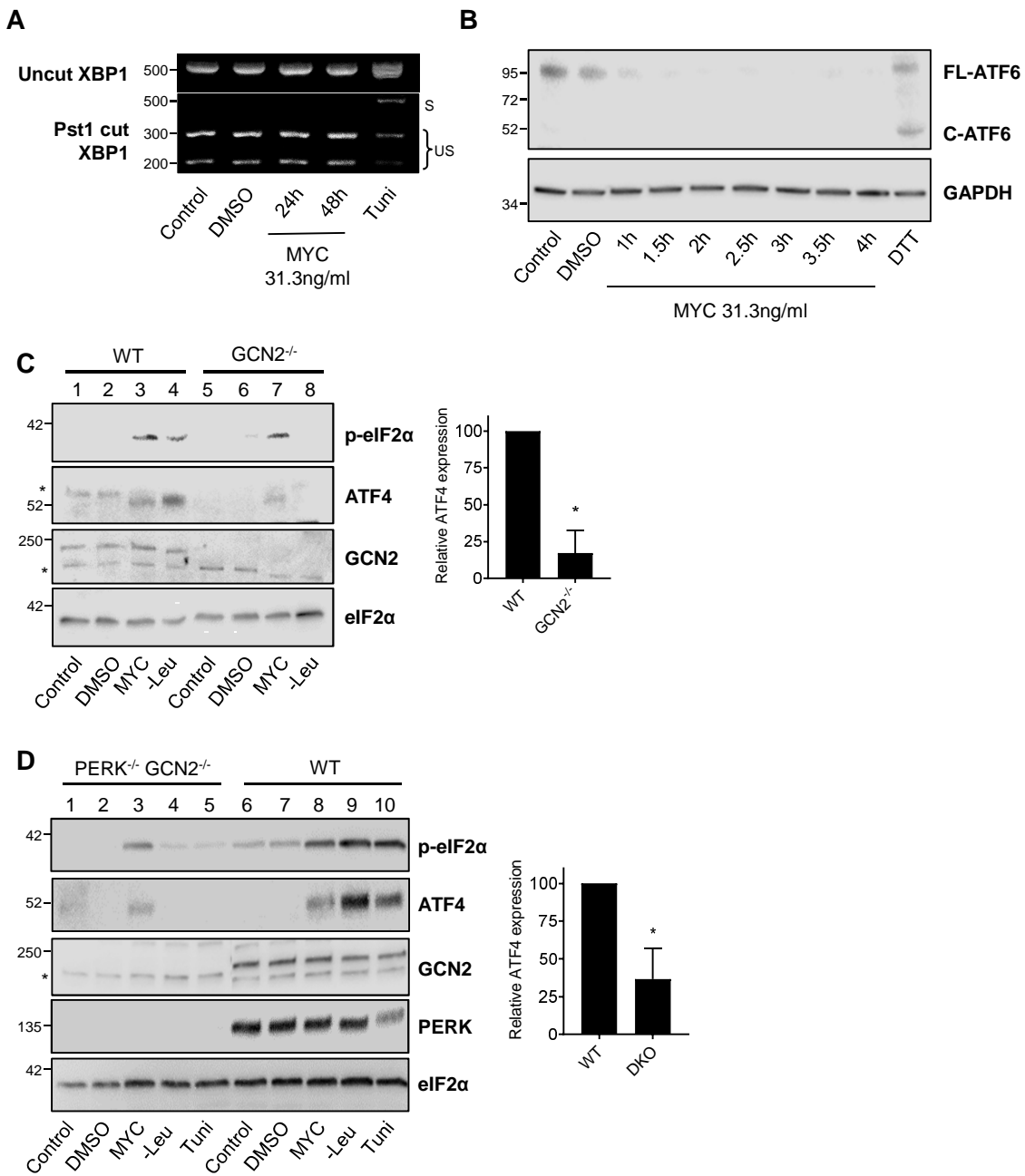

Fig. S3

Supplement: Supplementary file 6 — Figure S3(PDF 179 kb) [file 41419_2018_427_MOESM6_ESM.pdf]

**A**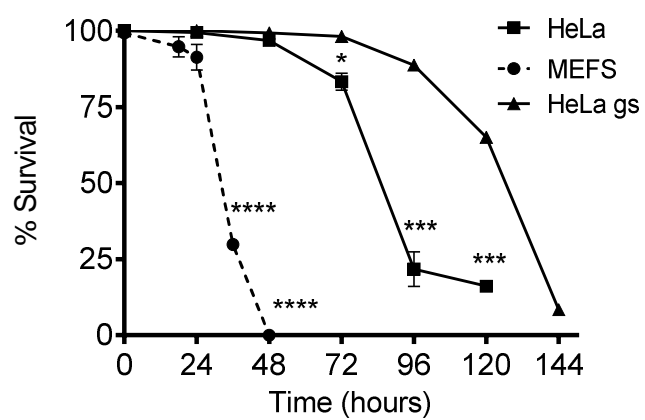**B**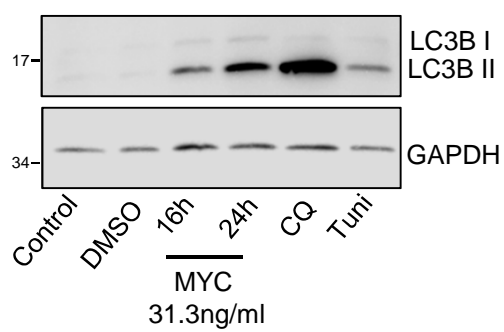

Fig. S4

Supplement: Supplementary file 7 — Figure S4(PDF 45 kb) [file 41419_2018_427_MOESM7_ESM.pdf]
