# Supplementary material for: Inhibition of Sec61-dependent translocation by mycolactone uncouples the integrated stress response from ER stress, driving cytotoxicity via translational activation of ATF4
Source: Cell Death Dis. 2018 Mar 14;9(3):397. doi: 10.1038/s41419-018-0427-y (PMC5852046; doi:10.1038/s41419-018-0427-y)
Supplement: Supplementary file 8 — Figure S5(PDF 15 kb) [file 41419_2018_427_MOESM8_ESM.pdf]

A

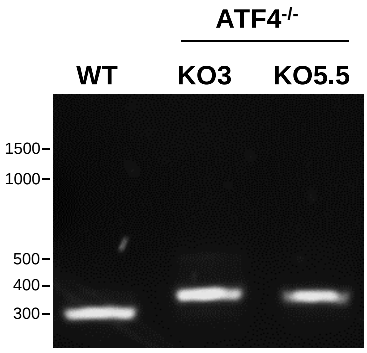

B

```
                                                     PAM
5′ TGCCCCCCTAGTCCAGGAGACTAATAAGCAGCCCCCCCAGACGGTGAACCCAATTGGCCATCTCCC 3′
   ||||||||||||||||||||||||||||||||||||||||||||||||||||||||||||||||||
3′ ACGGGGGGATCAGGTCCTCTGATTATTCGTCGGGGGGGTCTGCCACTTGGGTTAACCGGTAGAGGG 5′
         PAM
```

```
ID     SEQUENCE
    5′                                                                                                       3′
WT     TGCCCCCCTAGTCCAGGAGACTAATA           INS40NT                    AGCAGCCCCCCCAGACGGTGAACCCAATTGGCCATC
KO3    TGCCCCCCTAGTCCAGGAGACTAATAXXXXXXXXXXXXXXXXXXXXXXXXXXXCAGGAGACTAATAAGCAGCCCCCCCAGACGGTGAACCCAATTGGCCATC     ALLELE 1

WT     TGCCCCCCTAGTCCAGGAGACTAATA           INS43NT                        AGCAGCCCCCCCAGACGGTGAACCCAATTGGCCATC
KO3    TGCCCCCCTAGTCCAGGAGACTAATAXXXXXXXXXXXXXXXXXXXXXXXXXXXXXXXAGGAGACTAATAAGCAGCCCCCCCAGACGGTGAACCCAATTGGCCATC   ALLELE 2

WT     TGCCCCCCTAGTCCAGGAGACTAATA           INS41NT                     AGCAGCCCCCCCAGACGGTGAACCCAATTGGCCATC
KO5.5  TGCCCCCCTAGTCCAGGAGACTAATAXXXXXXXXXXXXXXXXXXXXXXXXXXXXCAGGAGACTAATAAGCAGCCCCCCCAGACGGTGAACCCAATTGGCCATC     ALLELE 1

WT     TGCCCCCCTAGTCCAGGAGACTAATA           INS44NT                         AGCAGCCCCCCCAGACGGTGAACCCAATTGGCCATC
KO5.5  TGCCCCCCTAGTCCAGGAGACTAATAXXXXXXXXXXXXXXXXXXXXXXXXXXXXXXXXCAGGAGACTAATAAGCAGCCCCCCCAGACGGTGAACCCAATTGGCCATC ALLELE 2
```

Fig. S5
